# Crack Contour Modeling Based on a Metaheuristic Algorithm and Micro-Laser Line Projection

**DOI:** 10.3390/biomimetics11020102

**Published:** 2026-02-02

**Authors:** J. Apolinar Muñoz Rodríguez

**Affiliations:** Centro de Investigaciones en Óptica, A. C., Lomas del Bosque 115, Col. Comas del Campestre, León 37000, GTO, Mexico; munoza@cio.mx

**Keywords:** crack contour model, metaheuristic genetic algorithm, Bezier basis function, micro-laser line scanning

## Abstract

Currently, bio-inspired metaheuristic algorithms play an important role in computer vision for assessing surface cracks. Also, manufacturing industries need non-destructive technologies based on biomimetics theory for characterizing micro-crack contours to determine surface quality. In this way, it is necessary to develop bio-inspired algorithms to construct crack contour models for determining crack regions through an optical microscope system. In this study, a metaheuristic genetic algorithm is implemented to build crack contour models by means of Bezier functions and crack coordinates. The contour modeling is performed by a microscope vision system based on micro-laser line scanning, which provides the crack coordinates through a broken laser line in the crack region. Thus, the metaheuristic algorithm builds the crack contour model by fitting the Bezier functions toward the crack topography. At this stage, an objective function moves the Bezier functions toward the crack topography via control points. The proposed technique provides micro-scale crack contours with a relative error smaller than 2%. Thus, the proposed crack contour modeling enhances the traditional crack contour inspection based on microscope image processing. This contribution is supported by a comparison between the proposed technique and the crack characterization performed via conventional image processing algorithms.

## 1. Introduction

Nowadays, computer vision algorithms have been employed to develop crack contour models for assessing surface quality in the manufacturing sector [1]. This mathematical modeling is a crucial process for determining crack regions on the surface being examined [2]. Thus, computer vision techniques have been employed for characterizing crack contours to quantify crack areas on a surface being inspected [3]. Hence, the crack areas are determined from the crack contours to assess the surface quality. This enables an effective surface crack inspection during the manufacturing process. Typically, the characterization of crack contours has been performed using computer vision methods such as filtering, segmentation, frequency domain, and deep learning. For instance, the characterization of crack contours has been deployed through Gaussian filtering and morphological operations [4], which identify crack regions through a contour fitting. Also, the characterization of crack contours has been carried out using homomorphic filtering that relies on a local threshold to establish the crack area [5], which is outlined by means of morphological operations and edge detection. Similarly, the crack contour has been defined by applying a Laplacian operator with a Gaussian smoothing filter [6], which determines the crack region by detecting high frequencies. Moreover, characterization of the crack contour has been developed via segmentation using image processing methods. In this context, the characterization of crack contours has been performed by means of segmentation using histogram equalization and binarization [7], which determines the region in which to perform a thinning for obtaining a crack skeleton. Also, the characterization of crack contours has been realized through segmentation utilizing an ortoboundary algorithm [8], which determines the crack area through the edge’s shortest distance and an orthogonal projection. Additionally, characterization of crack contours has been carried out using a segmentation network that relies on refined masks [9], which perform a semantic feature extraction to obtain crack edge details. In the same way, characterization of crack contours has been performed using skeleton techniques based on image processing. For example, characterization of crack contours has been carried out using a linear crack skeleton [10], which is fitted by a polynomial curve to determine the measurements of crack contours. Similarly, outlining of crack contours has been carried out using a linear crack skeleton with a flexible kernel of appropriate range [11], which extracts the skeleton from a division of the crack’s boundaries. Furthermore, characterization of crack contours has been implemented employing frequency domain methods. In this field, the characterization of crack contours has been effectuated using the Fourier transform [12], which determines discontinuities across the surfaces of the filled crack. Also, characterization of crack contours has been carried out using Fourier transform to detect planar defects [13], where the crack shape is determined based on a growth equation. Similarly, characterization crack of contours has been performed using multi-stage Fourier transform [14], which detects the crack surface through the secondary peaks in the Fourier transform spectrum. Recently, characterization of crack regions has been performed using deep learning methods via residual and convolutional neural networks. For instance, the modeling of crack contours has been carried out using a residual neural network [15], which learns from an image dataset and model hyper parameters to identify crack regions. Also, the modeling of crack contours has been performed through a residual neural network trained on ImageNet featuring cracks [16] and by computing a gradient-weighted class activation mapping. Similarly, the modeling of crack areas has been developed using a convolutional neural network focused on segmentation via crack images [17] and utilizes a stochastic gradient for learning to classify and locate crack areas. Also, crack modeling has been implemented using a convolutional neural network that relies on feature maps [18], where the training is effectuated with a crack dataset to identify cracks by means of feature maps.

The above-mentioned methods perform the modeling of crack contours using the distribution of pixel intensity. Nonetheless, the intensity profile does not accurately represent the crack topography. This is because the intensity distribution varies based on the surface reflectance, light source position, and viewer’s angle. These statements imply that conventional crack modeling techniques do not represent the crack profile through the real topography. This reduces accuracy in the modeling of crack contours. Additionally, deep learning methods build extensive models using a great number of images, resulting in reduced accuracy and slower performance [19]. This occurs because thousands of images are used to train the neural network models. Consequently, complex optimization must be performed to achieve the modeling of crack contours. Moreover, model optimization is carried out through additional parameters, resulting in supplementary procedures to obtain the crack contour model. On the other hand, simpler curve models such as polylines, B-splines, and clothoids have been used to build contour models. In this case, the polylines method fails to accurately represent contour curves [20], requiring a great number of lines to represent a contour curve. Also, the simpler B-splines do not intersect all points [21], requiring weights to achieve an accurate contour curve. Additionally, the clothoids curve is defined by cosine and sine functions [22], but an optimal curvature should be optimized to provide a contour curve. Furthermore, contour curves have been built using least squares to optimize Bezier functions [23], which do not pass through all points. Moreover, classical ridge/valley detectors have been employed to detect crack areas using image processing methods [24], which provide bi-dimensional information but low accuracy in three-dimensional coordinates. These criteria indicate that the modeling of crack contours remains a challenging endeavor. Consequently, it is essential to develop biomimetics theories for constructing crack contour models, emulating models of nature through metaheuristic algorithms and crack topography to improve crack assessment.

The proposed micro-scale crack contour modeling is performed using metaheuristic algorithms to determine crack areas, employing three-dimensional topography retrieved via laser line scanning. Thus, the crack contour model is developed by a metaheuristic algorithm employing Bezier functions and the coordinates of a broken laser line. Thus, the metaheuristic algorithm optimizes the Bezier functions using three-dimensional control points, which are computed by bio-inspired algorithms to generate crack contour models. Through this procedure, the crack contour model yields a three-dimensional Bezier curve, which depicts the crack contour area. The micro-scale crack contour modeling is carried out by an optical microscope vision system that includes a CCD camera and a 42 μm laser line. The microscope system is mounted on a slider device to move the vision system, which performs the scanning in the *x*-axis. Thus, the micro-laser line scans the surface, while the camera captures the broken laser line images to retrieve the crack contour region. In this process, the micro-scale topography is computed using the laser line position and the microscope’s geometry. Thus, the crack contour is determined using topography coordinates, providing high accuracy to improve the conventional crack inspection performed via image processing. In this context, the proposed technique provides crack contour measurements with a relative error smaller than 2%. Also, the proposed crack contour modeling can detect crack contours with a minimum surface width of 20 microns. This crack contour modeling improves the accuracy of the crack characterization via gray-scale image processing. The enhancement is achieved through the crack contouring performed by the micro-laser line projection. Also, the crack contour modeling approach improves the accuracy of measuring the crack region. This occurs due to the calculation of the Bezier basis functions by means of the three-dimensional crack topography. The contribution of crack contour modeling is established through a discussion focused on the accuracy of the crack characterization and detection via gray-scale image processing. The remainder of this paper is organized as follows: the basic theory for crack contour modeling using Bezier basis functions is described in Section 2.1, the crack contour modeling utilizing a metaheuristic algorithm based on Bezier basis functions is presented in Section 2.2, the recovering of crack surfaces through the broken laser line is detailed in Section 2.3, Section 2.4 covers the calibration of microscope parameters, the results of micro-surface crack modeling are presented in Section 3, and the contributions of the proposed micro-scale crack modeling are discussed in Section 4.

## 2. Materials and Methods

### 2.1. Basic Theory

**Figure 1:**
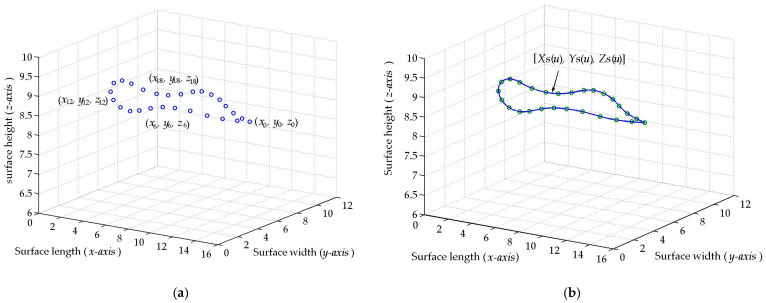
(**a**) Surface points to construct a crack contour model. (**b**) Crack contour generated by means of the Bezier curves in Equations (2)–(4).

The micro-scale crack contour modeling is performed using a metaheuristic algorithm and crack coordinates retrieved via micro-laser line scanning. In this context, the surface crack is defined as a deep surface discontinuity or fracture [25], where the surface material is absent. To determine a crack area, it is necessary to establish the crack contour, which defines the crack boundaries in a continuous form. Therefore, a mathematical model should be developed to represent the crack contour in continuous form. In this way, a metaheuristic algorithm is proposed to construct crack contour models by optimizing Bezier curves by means of control points. To carry it out, the algorithm performs explorations and exploitations to achieve optimal crack contour models. This procedure is carried out by minimizing an objective function, which is defined by means of Bezier functions and crack coordinates. Thus, the metaheuristic algorithm calculates the control points that move the Bezier curves toward the crack surface, generating crack contour models. In this way, the metaheuristic algorithm establishes the procedure for implementing crack contour models based on the coordinates of crack topography. The crack surface coordinates (*x_i_*, *y_j_*, *z_i_*) are illustrated in Figure 1a, where the subscript (*i*) indicates the number of the surface point in the contour topography. The coordinates of the crack surface are computed from a micro-laser line, which is broken in the crack region. Thus, the broken laser line yields the crack surface coordinates (*x*_0_, *y*_0_, *z*_0_), (*x*_1_, *y*_1_, *z*_1_), (*x*_2_, *y*_2_, *z*_2_), …, (*x_N_*, *y_N_*, *z_N_*), where the sub-index (*N*) indicates the total number of surface points in the crack topography. From these coordinates, the crack contour model is built by using the following fifth Bezier basis function:

(1) $$C_{s}(u)=\sum_{r=0}^{r=5}B_{r}(u)P_{r+5s},\;B_{r}(u)=\frac{5!}{r!(5-r)!}{\left(1-u\right)}^{5-r}u^{r},\;0\leq u\leq 1.$$

In this equation, *P_r_*_+5_*_s_* represents the control points that move the Bezier curve toward the position (*x_r_*_+5_*_s_*, *y_r_*_+5_*_s_*, *z_r_*_+5_*_s_*), where the subscript (*s*) indicates the number of the Bezier curve in the crack contour. From Equation (1), the following equations are obtained:

(2) $$X_{s}(u)=\sum_{r=0}^{r=5}B_{r}(u)P_{r+5s},\;\;u_{r}=\frac{\left(x_{r+5s}-x_{5s}\right)}{\left(x_{5(s+1)}-x_{5s}\right)},\;0\leq u\leq 1.$$

(3) $$Y_{s}(v)=\sum_{r=0}^{r=5}B_{r}(v)P_{r+5s},\;\;v_{r}=\frac{\left(y_{r+5s}-y_{5s}\right)}{\left(y_{5(s+1)}-y_{5s}\right)},\;0\leq v\leq 1.$$

(4) $$Z_{s}(w)=\sum_{r=0}^{r=5}B_{r}(w)P_{r+5s},\;\;w_{r}=\frac{\left(z_{r+5s}-z_{5s}\right)}{\left(z_{5(s+1)}-z_{5s}\right)},\;0\leq w\leq 1.$$

For these equations, the control points are defined by the expressions P*_r_*_+5_*_s_* = (*w_r_*_+5_*_s_*)*x_r_*_+5_*_s_*, P*_r_*_+5_*_s_* = (w*_r_*_+5_*_s_*)*y_r_*_+5_*_s_*, P*_r_*_+5_*_s_* = (*w_r_*_+5_*_s_*)*z_r_*_+5_*_s_*, where (*w_r_*_+5_*_s_*, w*_r_*_+5_*_s_*, *w_r_*_+5_*_s_*) are the weights that move the Bezier curve in *x*-direction, *y*-direction, and *z*-direction, respectively. Also, the sub-indices (*r*, *s*) are related to the sub-index (*i*) through the expression *i* = *r* + 5*s*, for *s* = 0, 1, 2,…, *M*. Thus, the crack contour model is represented by the curves [*X*_0_(*u*), *Y*_0_(*v*), *Z*_0_(*w*)], [*X*_1_(*u*), *Y*_1_(*v*), *Z*_1_(*w*)], [*X*_2_(*u*), *Y*_2_(*v*), *Z*_2_(*w*)], …, [*X_M_*(*u*), *Y_M_*(*v*), *Z_M_*(*w*)], where the sub-index (*M*) indicates the total number of Bezier curves in the crack contour model. The Bezier curves [*X_s_*(*u*), *Y_s_*(*v*), *Z_s_*(*w*)] of the crack points shown in Figure 1a are computed via Equations (2)–(4) to generate the crack contour illustrated in Figure 1b. These Bezier curves are determined by control points, which are computed using the weights (*w_r_*_+5_*_s_*, w*_r_*_+5_*_s_*, *w_r_*_+5_*_s_*). Thus, the crack contour model is defined by the following systems of equations:

(5) $$\left[\begin{array}{l} X_{0}(u)\\ X_{1,}(u)\\ X_{2,}(u)\\ \vdots \\ X_{M}(u) \end{array}\right]=\left[\begin{array}{l} B_{0}P_{0}+B_{1}P_{1}+B_{2}X_{2}+B_{3,}P_{3,}+B_{4,}P_{4}+B_{5,}P_{5}\\ B_{0}P_{5}+B_{1}P_{6}+B_{2}P_{7}+B_{3,}P_{8,}+B_{4,}P_{9}+B_{5,}P_{10}\\ B_{0}P_{10}+B_{1}P_{11}+B_{2}P_{12}+B_{3,}P_{13,}+B_{4,}P_{14}+B_{5,}P_{15}\\ \vdots \\ B_{0}P_{M-5}+B_{1}P_{M-4}+B_{2}P_{M-3}+B_{3,}P_{M-2,}+B_{4,}P_{M-1}+B_{5,}P_{M} \end{array}\right],$$

(6) $$\left[\begin{array}{l} Y_{0}(v)\\ Y_{1,}(v)\\ Y_{2,}(v)\\ \vdots \\ Y_{M}(v) \end{array}\right]=\left[\begin{array}{l} B_{0}P_{0}+B_{1}P_{1}+B_{2}P_{2}+B_{3,}P_{3,}+B_{4,}P_{4}+B_{5,}P_{5}\\ B_{0}P_{5}+B_{1}P_{6}+B_{2}P_{7}+B_{3,}P_{8,}+B_{4,}P_{9}+B_{5,}P_{10}\\ B_{0}P_{10}+B_{1}P_{11}+B_{2}P_{12}+B_{3,}P_{13,}+B_{4,}P_{14}+B_{5,}P_{15}\\ \vdots \\ B_{0}P_{M-5}+B_{1}P_{M-4}+B_{2}P_{M-3}+B_{3,}P_{M-2,}+B_{4,}P_{M-1}+B_{5,}P_{M} \end{array}\right],$$

(7) $$\left[\begin{array}{l} Z_{0}(w)\\ Z_{1,}(w)\\ Z_{2,}(w)\\ \vdots \\ Z_{M}(w) \end{array}\right]=\left[\begin{array}{l} B_{0}P_{0}+B_{1}P_{1}+B_{2}P_{2}+B_{3,}P_{3,}+B_{4,}P_{4}+B_{5,}P_{5}\\ B_{0}P_{5}+B_{1}P_{6}+B_{2}P_{7}+B_{3,}P_{8,}+B_{4,}P_{9}+B_{5,}P_{10}\\ B_{0}P_{10}+B_{1}P_{11}+B_{2}P_{12}+B_{3,}P_{13,}+B_{4,}P_{14}+B_{5,}P_{15}\\ \vdots \\ B_{0}P_{M-5}+B_{1}P_{M-4}+B_{2}P_{M-3}+B_{3,}P_{M-2,}+B_{4,}P_{M-1}+B_{5,}P_{M} \end{array}\right].$$

These systems of equations are solved to determine the weights (*w_r_*_+5_*_s_*, w*_r_*_+5_*_s_*, *w_r_*_+5_*_s_*), which yield the control points (P*_r_*_+5_*_s_*, P*_r_*_+5_*_s_*, P*_r_*_+5_*_s_*) for creating the crack contour model. Thus, the weights (*w_r_*_+5_*_s_*, w*_r_*_+5_*_s_*, *w_r_*_+5_*_s_*) are computed through a metaheuristic algorithm to build the crack contour model. In this way, the metaheuristic algorithm constructs a crack contour model using Bezier functions as shown in Figure 1b, where the Bezier curves [*X_s_*(*u*), *Y_s_*(*v*), *Z_s_*(*w*)] intersect all points on the crack surface.

**Figure 2:**
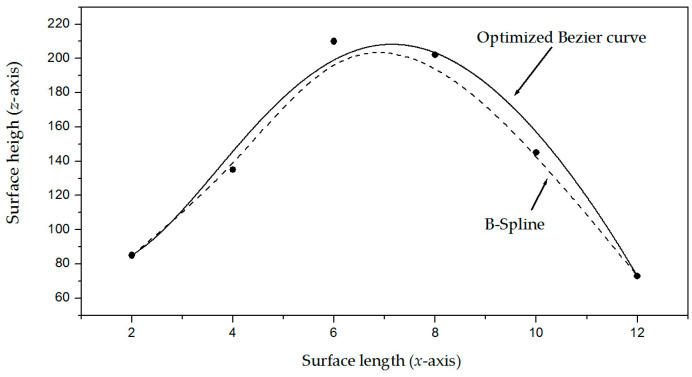
Cubic B-spline curve fitted from the surface points and Bezier curve generated via least squares.

The procedure to compute the weights (*w_r_*_+5_*_s_*, w*_r_*_+5_*_s_*, *w_r_*_+5_*_s_*) through the metaheuristic algorithm is described in Section 2.2, where the control points (P*_r_*_+5_*_s_*, P*_r_*_+5_*_s_*, P*_r_*_+5_*_s_*) are determined based on weights and crack surface coordinates. Thus, the metaheuristic algorithm determines the intervals of the weights (*w_r_*_+5_*_s_*, w*_r_*_+5_*_s_*, *w_r_*_+5_*_s_*) by utilizing the crack surface coordinates (*x_r_*_+5_*_s_*, *y_r_*_+5_*_s_*, *z_r_*_+5_*_s_*). This procedure moves the Bezier curves toward the crack surface via control points. Thus, the crack contour model is accomplished as shown in Figure 1b. To establish the viability of employing optimized Bezier curves to generate crack contour models, the features of the curves such as polylines, clothoids, B-splines, and Bezier curves are mentioned as follows. The polylines method builds a line segment for each surface point [26], requiring a great number of segments to generate a contour curve and reducing accuracy when the points are sampled. The clothoids method constructs smooth curve segments through cosine and sine functions, where the curvature is optimized to reduce the fitting error. The clothoids method is mathematically more complex and expensive compared to the Bezier curves [27]. The simpler B-splines method generates smooth curve segments providing continuity [28], but in some cases, the curve does not intersect all points. Consequently, an optimization of B-splines should be performed to improve accuracy. Moreover, the simpler Bezier curves have been optimized using least squares to reduce the fitting error [29]. To elucidate these criteria, a cubic B-spline curve and a Bezier curve optimized via least squares are fitted to a set of points. Figure 2 illustrates the result of the B-splines and the Bezier curve. The cubic B-splines method calculates curve segments through the points [30], which are indicated by the symbol • in Figure 2. In this figure, the outcome of the B-splines curve is represented by the dashed line. Similarly, the Bezier curve is computed using Equations (2) and (3), which are optimized through the least squares method [31] to determine the control points. The outcome of the Bezier curve generated via least squares is shown in Figure 2 by the solid line. As it is possible to see, the curves do not intersect all points. Consequently, the curve fitting can be improved to attain better accuracy. In this way, a metaheuristic algorithm is implemented to achieve an accurate crack contour model using Bezier curves. The implementation of the metaheuristic algorithm to generate a contour curve is described in Section 2.2.

### 2.2. Crack Contour Modeling via Metaheuristic Algorithm

The micro-sale crack contour model is generated through a metaheuristic algorithm and crack surface coordinates. In this way, the crack contour model is built by employing the crack coordinates (*x_i_*, *y_i_*, *z_i_*) shown in Figure 1a. Thus, the crack contour model is built by solving Equations (5)–(7) through the control points (P*_r_*_+5_*_s_*, P*_r_*_+5_*_s_*, P*_r_*_+5_*_s_*). In this way, the control points are computed via weights using the expressions P*_r_*_+5_*_s_* = (*w_r_*_+5_*_s_*)*x_r_*_+5_*_s_*, P*_r_*_+5_*_s_* = (w*_r_*_+5_*_s_*)*y_r_*_+5_*_s_*, P*_r_*_+5_*_s_* = (*w_r_*_+5_*_s_*)*z_r_*_+5_*_s_*. For calculating the weights, the crack coordinates and the parametric values (*u_i_*, *v_i_*, *w_i_*) are substituted in Equations (5)–(7) to obtain the following system of equations:

(8) $$\left[\begin{array}{l} X_{s}(u_{0})\\ X_{s,}(u_{1})\\ X_{s,}(u_{2})\\ \vdots \\ X_{s}(u_{5}) \end{array}\right]=\left[\begin{array}{llllll} B_{0}(u_{0}) & B_{1}(u_{0}) & B_{2}(u_{0}) & B_{3,}(u_{0}) & B_{4,}(u_{0}) & B_{5}(u_{0})\\ B_{0}(u_{1}) & B_{1}(u_{1}) & B_{2}(u_{1}) & B_{3,}(u_{1}) & B_{4,}(u_{1}) & B_{5}(u_{1})\\ B_{0}(u_{2}) & B_{1}(u_{2}) & B_{2}(u_{2}) & B_{3,}(u_{2}) & B_{4,}(u_{2}) & B_{5}(u_{2})\\ \vdots \\ B_{0}(u_{5}) & B_{1}(u_{5}) & B_{2}(u_{5}) & B_{3,}(u_{5}) & B_{4,}(u_{5}) & B_{5}(u_{5}) \end{array}\right]\left[\begin{array}{l} w_{9+5s}x_{9+5s}\\ w_{1+5s}x_{1+5s}\\ w_{2+5s}x_{2+5s}\\ w_{5+5s}x_{5+5s} \end{array}\right],$$

(9) $$\left[\begin{array}{l} Y_{s}(v_{0})\\ Y_{s,}(v_{1})\\ Y_{s,}(v_{2})\\ \vdots \\ Y_{s}(v_{5}) \end{array}\right]=\left[\begin{array}{llllll} B_{0}(v_{0}) & B_{1}(v_{0}) & B_{2}(v_{0}) & B_{3,}(v_{0}) & B_{4,}(v_{0}) & B_{5}(v_{0})\\ B_{0}(v_{1}) & B_{1}(v_{1}) & B_{2}(v_{1}) & B_{3,}(v_{1}) & B_{4,}(v_{1}) & B_{5}(v_{1})\\ B_{0}(v_{2}) & B_{1}(v_{2}) & B_{2}(v_{2}) & B_{3,}(v_{2}) & B_{4,}(v_{2}) & B_{5}(v_{2})\\ \vdots \\ B_{0}(v_{5}) & B_{1}(v_{5}) & B_{2}(v_{5}) & B_{3,}(v_{5}) & B_{4,}(v_{5}) & B_{5}(v_{5}) \end{array}\right]\left[\begin{array}{l} w_{0+5s}y_{0+5s}\\ w_{1+5s}y_{1+5s}\\ w_{2+5s}y_{2+5s}\\ w_{5+5s}y_{5+5s} \end{array}\right],$$

(10) $$\left[\begin{array}{l} Z_{s}(w_{0})\\ Z_{s,}(w_{0})\\ Z_{s,}(w_{2})\\ \vdots \\ Z_{s}(w_{5}) \end{array}\right]=\left[\begin{array}{llllll} B_{0}(w_{0}) & B_{1}(w_{0}) & B_{2}(w_{0}) & B_{3,}(w_{0}) & B_{4,}(w_{0}) & B_{5}(w_{0})\\ B_{0}(w_{1}) & B_{1}(w_{1}) & B_{2}(w_{1}) & B_{3,}(w_{1}) & B_{4,}(w_{1}) & B_{5}(w_{1})\\ B_{0}(w_{2}) & B_{1}(w_{2}) & B_{2}(w_{2}) & B_{3,}(w_{2}) & B_{4,}(w_{2}) & B_{5}(w_{2})\\ \vdots \\ B_{0}(w_{5}) & B_{1}(w_{5}) & B_{2}(w_{5}) & B_{3,}(w_{5}) & B_{4,}(w_{5}) & B_{5}(w_{5}) \end{array}\right]\left[\begin{array}{l} w_{0+5s}z_{0+5s}\\ w_{1+5s}z_{1+5s}\\ w_{2+5s}z_{2+5s}\\ w_{5+5s}z_{5r+5s} \end{array}\right].$$

These systems of equations are solved for *s* = 0, 1, 2, 3, …, *M* through the metaheuristic algorithm to obtain the weights (*w_r_*_+5_*_s_*, w*_r_*_+5_*_s_*, *w_r_*_+5_*_s_*), which generate the crack contour model [*X_s_*(*u*), *Y_s_*(*v*), *Z_s_*(*w*)]. Metaheuristics algorithms include genetic algorithms, which are implemented utilizing a random initial population, an objective function, an evaluation of the population’s fitness, selection of parents, a crossover to generate a new population, and a mutation in the new population [32,33]. Based on this conventional structure, a metaheuristic algorithm is implemented to compute the weights (*w_r_*_+5_*_s_*, w*_r_*_+5_*_s_*, *w_r_*_+5_*_s_*) that determine the control points of the crack contour model. To do so, the metaheuristic algorithm performs explorations and exploitations to find the optimal weights. In this way, the metaheuristic algorithm computes the weights in five steps, which are described as follows.

The first step computes Equations (2)–(4) using the control points P*_r_*_+5_*_s_* = *x_r_*_+5_*_s_*, P*_r_*_+5_*_s_* = *y_r_*_+5_*_s_*, P*_r_*_+5_*_s_* = *z_r_*_+5_*_s_* to determine the initial population of weights. Thus, if *Z_s_*(*w_r_*) exceeds the coordinate *z_r_*_+5_*_s_*, the upper limit for *w_r_*_+5_*_s_* is set at 1, while the lower limit for *w_r_*_+5_*_s_* is designated as 0.3. However, if *Z_s_*(*w_r_*) lies below the coordinate *z_r_*_+5_*_s_*, the minimum is set at 1, while the maximum is set at 1.7. Also, if *Y*_s_(*v_r_*) exceeds the coordinate *y_r_*_+5_*_s_*, the maximum w*_r_*_+5_*_s_* is set at 1, and the minimum w*_r_*_+5_*_s_* is set at 0.3. But, if *Y*_s_(*v_r_*) falls below the coordinate *y_r_*_+5_*_s_*, the minimum is set at 1, and the maximum is set at 1.7. Similarly, if *X*_s_(*u_r_*) exceeds the coordinate *x_r_*_+5_*_s_*, the maximum *w_r_*_+5_*_s_* is set at 1, and the minimum *w_r_*_+5_*_s_* is set at 0.3. But, if *X*_s_(*u_r_*) falls below the coordinate *x_r_*_+5_*_s_*, the minimum is specified as 1, whereas the maximum is set at 1.7. From this search space, the initial population is obtained by randomly selecting four values for each weight. These four values represent the parents (W_1,_*_k_*, W_2,_*_k_*, W_3,_*_k_*, W_4,_*_k_*) of each weight, with the *k*-index indicating the generation number. Thus, the initial population is established. Also, the search space provides all necessary weights to determine the optimal control points. This is because the solution space includes weights that generate curves both above and below the surface points. Additionally, the population’s size is bigger and encompasses all candidates from the search space. This is because a new parent is randomly tested from the search space during the iteration of the algorithm. In this context, the size of the population is unlimited, and the potentials candidates are not eliminated.

The second step creates the *k*-generation children via crossover by computing explorations and exploitations [34]. This procedure provides two children within parents and one child outside parents. Thus, the children (C_1,_*_k_*, C_2_*_,k_*) and (C_4,_*_k_*, C_5_*_,k_*) are created by means of explorations from the parents (W_1,_*_k_*, W_2,_*_k_*) and (W_3,_*_k_*, W_4,_*_k_*), respectively. Additionally, the children (C_3,_*_k_*, C_6_*_,k_*) are created by means of exploitations. In this way, the current children are determined by computing the following equations:

(11) $$C_{1,k}=0.5\left(W_{1,k}+W_{2,k}\right)+0.5\beta \left|W_{1,k}-W_{2,k}\right|,$$

(12) $$C_{2,k}=0.5\left(W_{1,k}+W_{2,k}\right)-0.5\beta \left|W_{1,k}-W_{2,k}\right|,$$

(13) $$C_{4,k}=0.5\left(W_{4,k}+W_{3,k}\right)+0.5\beta \left|W_{4,k}-W_{3,k}\right|,$$

(14) $$C_{5,k}=0.5\left(W_{4,k}+W_{3,k}\right)-0.5\beta \left|W_{4,k}-W_{3,k}\right|,$$

(15) $$C_{3,k}=W_{0,k}+\beta \left|W_{1,k}-W_{0,k}\right|,$$

(16) $$C_{6,k}=W_{4,k}+\beta \left|W_{5,k}-W_{4,k}\right|.$$

For these equations, W_0,_*_k_* and W_5,_*_k_* represent the minimum and maximum of each weight, respectively. The parameter *β* is calculated through a factor *α*, which is randomly selected from the interval between 0 and 1. Thus, *β* = (2*α*)^1/2^ if *α* > 0.5; otherwise, *β* = [2(1 − *α*)]^1/2^. In this way, Equations (11)–(14) compute the children within parents, while Equations (15) and (16) compute the children external to parents. From these criteria, the *k*-generation children are computed. The third step evaluates the population’s fitness through an objective function, which is represented by the following expression:

(17) $$O_{s}=\min\left\{\sqrt{\frac{1}{6}\sum_{r=0}^{r=5}[{\left(X_{s}(u_{r})-x_{r+5s}\right)}^{2}+{\left(Y_{s}(u_{r})-y_{r+5s}\right)}^{2}+{\left(Z_{s}(u_{r})-z_{r+5s}\right)}^{2}]}\right\}.$$

The fourth step takes the best current parents and children to select the (*k* + 1)-generation parents. Thus, the parent W_1,_ *_k_*_+1_, is obtained from (W_1,_*_k_*, W_2,_*_k_*), the parent W_3,_*_k_*_+1_ is gathered from (W_3,_*_k_*, W_4,_*_k_*), the parent W_2,_*_k_*_+1_ is taken from (C_1,_*_k_*, C_2,_*_k_*, C_3,_*_k_*), and the parent W_4,_*_k_*_+1_ is selected from (C_4,_*_k_*, C_5,_*_k_*, C_6,_*_k_*).

The fifth step mutates one parent and one weight to avoid a local minimum. To do so, a new parent takes the place of the worst parent to evaluate the fitness according to Equation (17). If the new parent enhances the fitness, it replaces the worst parent. Otherwise, the mutation does not occur. Additionally, a new weight randomly replaces a weight from a parent that is chosen in random form. Thus, if the updated weight improves the fitness, the new weight will replace the chosen weight. If not, the chosen weight remains unchanged. Also, the (*k* + 1)-generation children are generated by computing Equations (11)–(16). Based on these statements, steps two through five are iteratively computed to obtain the optimal weights that minimize the objective function Equation (17).

**Figure 3:**
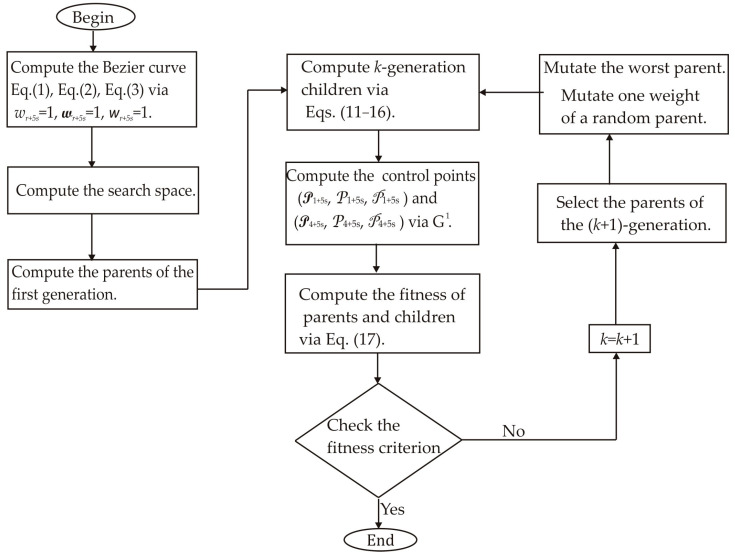
Flowchart of the metaheuristic algorithm to compute the control points of the crack contour model.

To elucidate the stages of the metaheuristic algorithm, the crack contour [*X*_0_(*u*), *Y*_0_(*v*), *Z*_0_(*w*)] is computed from the crack surface points illustrated in Figure 1a. In this way, the control points (P_0+5s_, P_0+5s_, P_0+5s_), (P_1+5s_, P_1+5s_, P_1+5s_), (P_2+5s_, P_2+5s_, P_2+5s_), …, (P_5+5s_, P_5+5s_, P_5+5s_) are calculated using the metaheuristic algorithm sketched in the flowchart shown in Figure 3. For a Bezier curve [*X_s_*(*u*), *Y_s_*(*v*), *Z_s_*(*w*)], the control points are defined as P_0_*_s_* = *x*_5_*_s_*, P_0_*_s_* = *y*_5_*_s_*, P_5_*_s_* = *z*_0_*_s_*, P_5_*_s_* = *x*_5_*_s_*, P_5_*_s_* = *y*_5_*_s_*, P_5_*_s_* = *z*_5_*_s_*, while the weights are set at *w*_0_*_s_* = 1, w_0_*_s_* = 1, *w*_0_*_s_* = 1, *w*_5_*_s_* = 1, w_5_*_s_* = 1, *w*_5_*_s_* = 1. Additionally, the control points (P_1+5_*_s_*, P_1+5_*_s_*, P_1+5_*_s_*), and (P_4+5__*s*_, P_4+5_*_s_*, P_4+5_*_s_*) are determined by the expressions P_1+5_*_s_* = (*x*_0+5_*_s_* +*x*_1+4_*_s_*)/2, P_1+5_*_s_* = (*y*_0+5_*_s_* + *y*_1+4_*_s_*)/2, P_1+5_*_s_* = (*z*_0+4_*_s_* + *z*_1+4_*_s_*)/2, P_4+5_*_s_* = (*x*_3+4_*_s_* + *x*_4+4_*_s_*)/2, P_4+5_*_s_* = (*y*_3+4_*_s_* + *y*_4+4_*_s_*)/2, P_4+5_*_s_* = (*z*_3+4_*_s_* + *z*_4+4_*_s_*)/2 to provide continuity *G*^1^. According to these criteria, the control points (P_2+5s_, P_2+5s_, P_2+5s_), (P_3+5s_, P_3+5s_, P_3+5s_) are computed by the metaheuristic algorithm as follows.

The first step computes Equations (2)–(4) using the weights *w_r_*_+5_*_s_* = 1, w*_r_*_+5_*_s_* = 1, *w_r_*_+5_*_s_* = 1*_s_* to determine the initial population of each weight. Thus, if *Z*_s_(*u_r_*) exceeds *z_r_*_+5_*_s_*, the upper and lower limits for *w_r_*_+5_*_s_* are set at 1 and 0.3, respectively. But, if *Z*_s_(*u_r_*) falls under *z_r_*_+5_*_s_*, the maximum and minimum are defined as 1.7 and 1. Also, if *Y*_s_(*u_r_*) exceeds *y_r_*_+5_*_s_*, the maximum and the minimum values of w*_r_*_+5_*_s_* are set at 1 and 0.3, respectively. But, if *Y*_s_(*u_r_*) is under *y_r_*_+5_*_s_*, the maximum and minimum are set at 1.7 and 1. Likewise, if *X*_s_(*u_r_*) exceeds *x_r_*_+5_*_s_*, the maximum and minimum values of *w_r_*_+5_*_s_* are defined as 1 and 0.3. However, if *X*_s_(*u_r_*) is under *x_r_*_+5_*_s_*, the maximum and minimum values are set at 1.7 and 1. From this solution space, four parents (W_1,1_, W_2,1_, W_3,1_, W_4,1_) are randomly chosen to generate the initial population for every weight. Table 1 illustrates these initial parents, with the control points to be calculated in the first column and the parents identified in the second through fifth columns. The second step computes Equations (11)–(16) to generate the current children (C_1,_*_k_*, C_2,_*_k_*, C_3,_*_k_*, C_4,_*_k_*, C_5,_*_k_*, C_6,_*_k_*) via crossover. Also, the control points P_1+5_*_s_* = (*x*_0+5_*_s_* + *x*_1+4_*_s_*)/2, P_1+5_*_s_* = (*y*_0+5_*_s_* + *y*_1+4_*_s_*)/2, P_1+5_*_s_* = (*z*_0+4_*_s_* + *z*_1+4_*_s_*)/2, P_4+5_*_s_* = (*x*_3+4_*_s_* + *x*_4+4_*_s_*)/2, P_4+5_*_s_* = (*y*_3+4_*_s_* + *y*_4+4_*_s_*)/2, P_4+5_*_s_* = (*z*_3+4_*_s_* + *z*_4+4_*_s_*)/2 are computed to provide continuity *G*^1^. Thus, the first children are computed for *k* = 1, and they are shown in the sixth through eleventh columns of Table 1. Next, the third step computes the fitness by substituting [*X_s_*(*u_r_*), *Y_s_*(*v_r_*), *Z_s_*(*w_r_*)] and [*x_r_*, *y_r_*, *z_r_*] into the objective function Equation (17) for *s* = 0 and *r* = 0, 1, 2, 3, 4, 5. The fitness of the first population is indicated in the seventh row of Table 1. This fitness shows that the initial population provides a small error after the first generation. Then, the fourth step selects the (*k* + 1)-generation parents from the best current parents and children. In this way, the parent W_1,_*_k_*_+1_ is selected from (W_1,_*_k_*, W_2,_*_k_*), the parent W_3,_*_k_*_+1_ is gathered from (W_3,_*_k_*, W_4,_*_k_*), the parent W_2,_*_k_*_+1_ is sourced from (C_1,_*_k_*, C_2,_*_k_*, C_3,_*_k_*), and the parent W_4,_*_k_*_+1_ is chosen from (C_4,_*_k_*, C_5,_*_k_*, C_6,_*_k_*). Thus, W_1,2_ = W_1,1_, W_2,2_ = C_2,1_, W_3,2_ = W _4,1_, W_4,2_ = C_5,1_ are obtained.

Following this, the fifth step mutates the worst parent W_3,2_, which is selected via fitness. Thus, a new parent takes the place of the worst parent to evaluate Equation (17). In this case, the fitness has been improved, so the new parent replaces the parent W_3,2_. Also, the parent W_1,2_ is randomly selected to mutate the weight *w*_3,2_, which is chosen in random form. Thus, a new weight substitutes the weight *w*_3,2_ to evaluate the fitness. In this case, the new weight enhances the fitness, so the new weight replaces the weight *w*_3,2_. Next, the second step computes Equations (11)–(16) to generate the second children. Additionally, the control points P_1+5_*_s_* = (*x*_0+5_*_s_* + *x*_1+4_*_s_*)/2, P_1+5_*_s_* = (*y*_0+5_*_s_* + *y*_1+4_*_s_*)/2, P_1+5_*_s_* = (*z*_0+4_*_s_* + *z*_1+4_*_s_*)/2, P_4+5_*_s_* = (*x*_3+4_*_s_* + *x*_4+4_*_s_*)/2, P_4+5_*_s_* = (*y*_3+4_*_s_* + *y*_4+4_*_s_*)/2, P_4+5_*_s_* = (*z*_3+4_*_s_*+ *z*_4+4_*_s_*)/2 are calculated to provide continuity *G*^1^. Also, the fitness of the second children is computed via Equation (17). Table 2 illustrates the second-generation population.

The process to compute the (*k* + 1)-generation population is iteratively computed to minimize the objective function Equation (17). Thus, the weights are obtained to determine the optimal control points, which are indicated in the twelfth column of Table 2. These control points are used to compute the crack contour curve [*X*_0_(*u*), *Y*_0_(*v*), *Z*_0_(*w*)] shown in Figure 1b. Similarly, the contour curves [*X*_1_(*u*), *Y*_1_(*v*), *Z*_1_(*w*)], [*X*_2_(*u*), *Y*_2_(*v*), *Z*_2_(*w*)], …, [*X_M_*(*u*), *Y_M_*(*v*), *Z_M_*(*w*)] are computed to obtain the crack contour illustrated in Figure 1b. In this way, the crack contour model has been built. Section 2.3 describes the detection of crack surface through micro-laser line scanning.

### 2.3. Crack Detection via Micro-Laser Line Scanning

**Figure 4:**
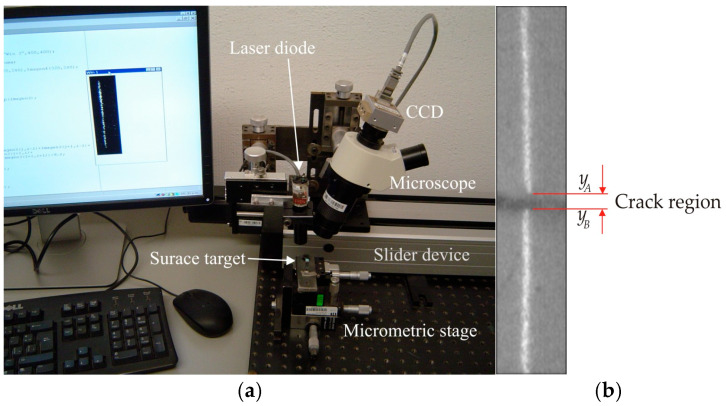
(**a**) Microscope vision system to retrieve micro-scale crack contour. (**b**) Micro-laser line projected on a crack surface.

The micro-scale crack contour model is constructed employing crack surface coordinates retrieved via micro-laser line scanning. Thus, the laser line scanning is carried out by the microscope vision system shown in Figure 4a. This microscope system includes a micro-laser line and a CCD array, which are connected to the optical microscope. Additionally, the microscope system is mounted on a slider device, which moves the arrangement to perform laser line scanning. In this microscope system, the *x*-axis denotes the horizontal axis, the *y*-axis depicts the depth, and the *z*-axis indicates the vertical axis. Thus, the microscope system perpendicularly projects the micro-laser line on the surface, while the CCD camera captures the laser line reflection to detect the surface crack. In this context, the surface crack is defined as a deep surface discontinuity or a material’s fracture. Additionally, the laser line is broken in the crack area due to the significant surface discontinuity. this is because a fracture does not reflect the laser line due to the absence of the surface. These criteria are elucidated by the broken laser line illustrated in Figure 4b, which has been captured from a surface crack. In this case, the broken laser line indicates the surface crack region from *y*_A_ to *y*_B_ along the *y*-axis. Also, the crack contour area is established in the *x*-axis and *y*-axis, while the crack contour height is defined in the *z*-direction. Thus, the surface crack coordinates are computed based on the position of the broken laser line. To do this, the surface is scanned in order to compute the crack coordinates by using the laser line position and the microscope’s geometry.

**Figure 5:**
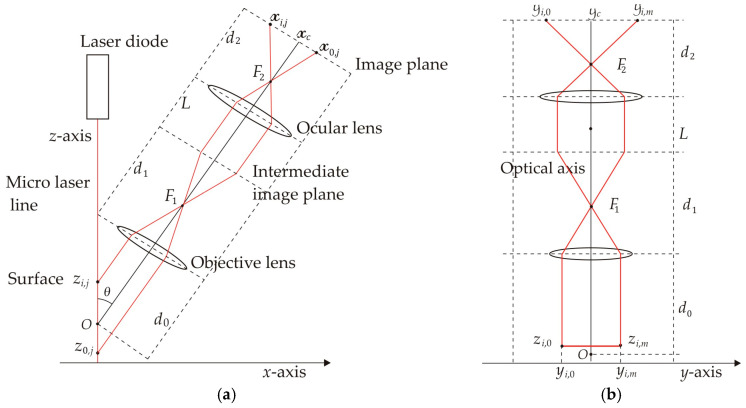
(**a**) Lateral geometry of the microscope system in *x*-axis. (**b**) Geometry of the microscope system in *y*-axis.

The crack coordinates in the *z*-axis are deduced from the microscope geometry in the *x*-direction, which is illustrated in Figure 5a, where a 42 μm laser line is perpendicularly projected on the surface, which reflects the laser line onto the CCD array through the microscope. In this geometry, the symbol *θ* denotes the angle formed by the laser line and the optical axis; the length *d*_0_ depicts the distance from the topography point *O* to the objective lens; the length *d*_1_ represents the distance from the intermediate plane to the first objective lens, while *F*_1_ denotes the focal position of the objective lens. The length *L* depicts the distance from the ocular lens to the intermediate image plane; the length *d*_2_ represents the distance from the CCD array to the ocular lens, while *F*_2_ indicates the focal position of the ocular lens. The microscope’s lateral geometry in the *y*-axis is depicted in Figure 5b. Additionally, the location of the laser line in the image plane is represented by (x_i,j_, y_i,j_); the image center is denoted by (x_c_, y_c_); and the pixel size is represented by the symbol *η*.

The surface height *z_i_*_,_*_j_* and the surface width *y_i_*_,_*_j_* are calculated from the geometry depicted in Figure 5a,b by means of the following expressions:

(18) $$z_{i,j}=\frac{\eta (x_{i,j}-x_{c})F_{1}F_{2}}{(F_{1}-d_{1})(d_{2}-F_{2})sin\theta }+O,$$

(19) $$y_{i,j}=\frac{\eta (y_{i,m}-y_{c})F_{1}F_{2}}{\left(F_{1}-d_{1})(d_{2}-F_{2}\right)}+\eta y_{c}.$$

From these equations, the surface height *z_i_*_,_*_j_* and the surface width *y_i_*_,_*_j_* are computed by means of the parameters (x_c_, y_c_, *η*, *θ*, *d*_1_, *F*_1_, *d*_2_, *F*_2_). But, the surface coordinate *x_i_*_,_*_j_* is provided by the slider device. In this way, the microscope vision system scans the target surface to determine the laser line coordinates (x*_i_*_,_*_j_*, y*_i_*_,_*_j_*) from the image. Thus, the surface depth *z_i_*_,_*_j_* is computed by substituting x*_i_*_,_*_j_* in Equation (18), while the surface width *y_i_*_,_*_j_* is calculated by replacing y*_i_*_,_*_j_* in Equation (19). In the image plane, the laser line coordinates (x*_i_*_,_*_j_*, y*_i_*_,_*_j_*) are determined by detecting the maximum intensity in each row of the image. Thus, the laser line detection is performed by computing the intensity maximum sum of five pixels in each row. This summation includes the maximum pixel, two pixels to the left of the maximum, and two pixels to the right of the maximum. Thus, if the intensity sum exceedes 680, the laser line exists. But, if the intensity summation is smaller than 680, the laser line is nonexistent. In this case, a crack region is identified, and the position of the laser line is not computed. Thus, the laser line coordinate x_i,j_ is calculated through the maximum intensity in the *x*-axis [35]. To do this, the laser line intensity is fitted to a Bezier curve in the *x*-direction by means of the following expressions:

(20) $$x(u)=\sum_{i=0}^{N}C_{i}{\left(1-u\right)}^{N-i}u^{i}x_{i,j},\;\;\;C_{i}=C_{i-1}(N+1-i)/i,\;\;C_{0}=1,\;\;\;0\leq u\leq 1.$$

(21) $$I(u)=\sum_{i=0}^{N}C_{i}{\left(1-u\right)}^{N-i}u^{i}I_{i,j},\;\;\;C_{i}=C_{i-1}(N+1-i)/i,\;\;C_{0}=1,\;\;\;0\leq u\leq 1.$$

For these equations, x*_i_*_,_*_j_* denotes the pixel position of the laser line along the *x*-axis, *I_i_*_,_*_j_* represents the pixel intensity, and *N* indicates the number of pixels of the laser line width in the *x*-axis. In this case, the sub-indices (*i*, *j*) represent the pixel number in the *x*-axis and *y*-axis, respectively. For calculating the Bezier curve, the pixel location x*_i_*_,_*_j_* and the intensity *I_i_*_,_*_j_* are substituted in Equations (20) and (21), respectively. By computing these equations, a concave curve [x(*u*), *I*(*u*)] is obtained within the interval 0 ≤ *u* ≤ 1, where the second derivative *I*”(*u*) is positive. In this way, the maximum intensity is determined by computing the first derivative *I*’(*u*) = 0, where *u* is calculated using the Bisection method. Thus, the value *u* is substituted in Equation (20) to calculate x(*u*), which represents the laser line position x*_i_*_,_*_j_* = x(*u*) in the *x*-axis. On the other hand, the laser line coordinate y*_i_*_,_*_j_* is obtained from the row number in the *y*-axis. Additionally, the laser line edges y*_i_*_,0_ and y*_i_*_,_*_m_* are determined by calculating the first derivative in the *y*-axis. In this case, the sub-index (*m*) indicates the total number of rows of the laser line in the *y*-axis. Thus, the coordinate x*_i_*_,_*_j_* is substituted in Equation (18) to calculate the surface height *z_i_*_,_*_j_*, while the coordinate y*_i,j_* is replaced in Equation (19) to determine the surface width *y_i_*_,_*_j_*. Through this procedure, the surface height *z_i_*_,_*_j_* and the surface width *y_i_*_,_*_j_* are computed from the laser line image, while the surface coordinate *x_i_*_,_*_j_* is given by the slider mechanism. Consequently, the crack contour position is determined from the beginning and end of the broken laser position on the *y*-axis. In this way, the crack contour coordinates are acquired to compute the crack contour model using the metaheuristic algorithm described in Section 2.2. The procedure to compute the parameters of the microscope vision system is described in Section 2.4.

### 2.4. Vision Parameters of the Optical Microscope Vision System

**Figure 6:**
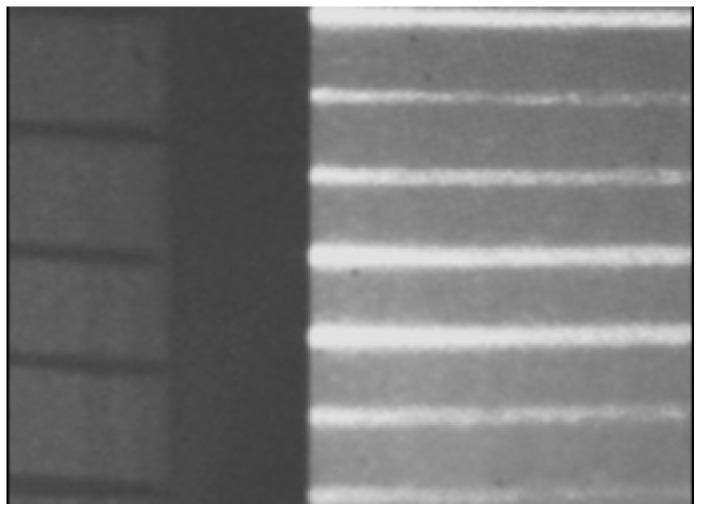
Line pattern with known dimensions to compute microscope parameters.

The surface depth *z_i_*_,_*_j_* and the surface width *y_i_*_,_*_j_* are determined through the microscope parameters (x*_c_*, y*_c_*, *η*, *θ*, *d*_1_, *d*_2_, *F*_1_, *F*_2_). These parameters are calculated through a metaheuristic algorithm employing Equations (18) and (19) to deduce the objective function. To do this, the algorithm uses the known surface height (*z_i_*_,_*_j_*–*z*_0,_*_j_*) and the known surface width (*y_i_*_,_*_j_*–*y_i_*_,_*_m_*). In context, the surface width (*y_i_*_,_*_j_*–*y_i_*_,_*_m_*) is obtained by employing a line pattern with known dimensions in the *y*-axis. This line pattern is shown in Figure 6, where the scale is indicated in millimeters in the *y*-axis. In this case, the line pattern is placed at the position *z*_0,_*_j_* in the *z*-axis. For this line pattern, the position of each line is indicated by y*_i_*_,0_, y*_i_*_,1_, y*_i_*_,2_, …, y*_i_*_,_*_m_* in the image plane. These line positions are determined by computing the maximum intensity through the Bezier curves Equations (20) and (21), where *x_i,j_* is obtained from the line pixel position along the *y*-axis and y*_i_*_,_*_j_* = x(*u*). Based on the line positions, the surface width (*y_i_*_,_*_j_*–*y_i_*_,_*_m_*) is obtained for *j* = 0, 1, 2, 3, …, *m*. Then, the line pattern is moved at the position *z*_0,_*_j_* in the *z*-axis by means of the micrometric stage, which is shown in Figure 4a. Thus, the micrometric stage provides the surface positions *z_i_*_,_*_j_* and *z*_0,_*_j_* in the *z*-axis. In this way, surface height (*z_i_*_,_*_j_*–*z*_0,_*_j_*) is obtained to determine the microscope vision parameters by means of the metaheuristic algorithm. The implementation of the metaheuristic algorithm is performed by the following steps. The first step calculates the search space and the initial population for every parameter. The search space of the parameters (x*_c_,* y_c_*, η*) is deduced from the image dimensions. But, the search space of the parameters (*d*_1_, *F*_1_, *d*_2_, *F*_2_, *θ*) is determined based on the microscope geometry illustrated in Figure 5a. Thus, the minimum *F*_2_ is deduced by multiplying the ocular lens ratio by 1.3, while the maximum *F*_2_ is determined by multiplying the ocular lens ratio by 2.3.

Similarly, the minimum *d*_2_ is defined by multiplying the ocular lens ratio by 1.4, while the maximum *d*_2_ is deduced by multiplying the ocular lens ratio by 2.8. Also, the minimum and maximum values of *F*_1_ are determined by multiplying the objective lens ratio by 1.3 and 2.3, respectively. Additionally, the minimum and maximum values of *d*_1_ are deduced by multiplying the objective lens ratio by 1.4 and 3.2, respectively. The minimum and maximum values of *θ* are set at 15° and 50°, respectively. Then, four parents (W_1,_*_k_*, W_2,_*_k_*, W_3,_*_k_*, W_4,_*_k_*) are randomly selected from the limits of each parameter’s range. Thus, the initial population of the parameters (*x_c_*, *y_c_*, *η*, *θ*, *d*_1_, *F*_1_, *d*_2_, *F*_2_) are established. The second step calculates Equations (11)–(16) to create the children (C_1,_*_k_*, C_2,_*_k_*, C_3,_*_k_*, C_4,_*_k_*, C_5,_*_k_*, C_6,_*_k_*). Next, the third step evaluates the fitness using the following expressions:

(22) $$O_{1}=\min\left\{\frac{1}{mxn}{\sum_{i=}^{n}\sum_{j=0}^{m}\left[(z_{i,j}-z_{0,j})-\frac{\eta (x_{c}-x_{i,j})F_{1}F_{2}}{(d_{1}d_{2}-d_{1}F_{2}-d_{2}F_{1}+F_{1}F_{2})sin\theta }+\frac{\eta (x_{c}-x_{i,m})F_{1}F_{2}}{(d_{1}d_{2}-d_{1}F_{2}-d_{2}F_{1}+F_{1}F_{2})sin\theta }\right]}^{2}\right\},$$

(23) $$O_{2}=\min\left\{\frac{1}{mxn}{\sum_{i=}^{n}\sum_{j=0}^{m}\left[(y_{i,m}-y_{i,0})+\frac{\eta (y_{c}-y_{i,j})F_{1}F_{2}}{\left(d_{1}d_{2}-d_{1}F_{2}-d_{2}F_{1}+F_{1}F_{2}\right)}-\frac{\eta (y_{c}-y_{i,m})F_{1}F_{2}}{\left(d_{1}d_{2}-d_{1}F_{2}-d_{2}F_{1}+F_{1}F_{2}\right)}\right]}^{2}\right\}.$$

From these equations, the fitness is computed by the expression *O* = (*O*_1_ + *O*_2_)/2, where the data (*z_i_*_,_*_j_*–*z_i_*_,_*_m_*) and (*y_i_*_,_*_j_*–*y_i_*_,_*_m_*) are known. Then, the fourth step selects the parents for the (*k* + 1)-generation. Thus, the parents W_1,k+1_ and W_3,k+1_ are selected from the parents (W_1,k_, W_2,k_) and (W_3,k_, W_4,k_). But, the parents W _2,_*_k_*_+1_ and W_4,_*_k_*_+1_ are chosen from the children (C_1,_*_k_*, C_2,_*_k_*, C_3,__*k*_) and (C_4,_*_k_*, C_5,_*_k_*, C_6,_*_k_*), respectively. Then, the fifth step replaces the worst parent with a new parent. Thus, if the new parent improves the fitness, the worst parent undergoes mutation. If not, the worst parent remains unchanged. Also, a new parameter replaces a parameter that is randomly selected. Thus, if the new parameter improves the fitness, the parameter undergoes mutation. If not, the parameter remains unchanged. In this way, the (*k* + 1)-generation parents are acquired. Also, the (*k* + 1)-generation children are generated by computing Equations (11)–(16) to obtain the (*k* + 1)-generation population. The procedure to determine the (*k* + 1)-generation population is iteratively computed to find the parameters (*x_c_*, *y_c_*, *η*, *θ*, *d*_1_, *F*_1_, *d*_2_, *F*_2_) that minimize Equations (22) and (23). Additionally, the distance between zero and the point *O* is determined by computing the expression *z*_0,_*_j_* = *η*(x_0_*_,j_* − x*_c_*) *F*_1_*F*_2_/(*d*_1_ − *F*_1_)(*d*_2_ − *F*_2_)*sin θ*. Thus, the metaheuristic algorithm has computed the vision parameters (*x_c_*, *y_c_*, *η*, *θ*, *d*_1_, *F*_1_, *d*_2_, *F*_2_), obtaining the next results: *d*_1_ = 62.361 mm, *F*_1_ = 30.689 mm, *d*_2_ = 60.253 mm, *F*_2_ = 27.403 mm, *θ* = 22.431°, *x_c_* = 395.96 pixels, *y_c_* = 373.36 pixels, *η* = 2.22 microns/pixel. In this procedure, the metaheuristic algorithm was computed 110 times to determine the uncertainties of the microscope parameters (*x_c_*, *y_c_*, *η*, *θ*, *d*_1_, *F*_1_, *d*_2_, *F*_2_) by means of the standard deviation. To do this, the coordinates (x*_i_*_,_*_j_*, y*_i_*_,_*_j_*) computed from (*z_i_*_,_*_j_*–*z*_0,_*_j_*) and (*y_i_*_,_*_j_*–*y_i,m_*) should be the same for each time that the algorithm computes the microscope parameters. Thus, the same values of (*z_i_*_,_*_j_*–*z*_0,_*_j_*) and (*y_i_*_,_*_j_*–*y_i,m_*) are employed for each time that the parameters are computed. In this way, the algorithm computes the uncertainty of vision parameters via standard deviation. The values of the microscope vision parameters and the variables of crack contour modeling are described in Table 3, where the first column depicts the symbol of every variable of the microscope system and the crack contour modeling; the second column represents the units of each variable; the values of the microscope vision parameters are shown in the third column; the vision parameters uncertainty is pointed in the fourth column; the values of the microscope vision parameters with uncertainty are represented in the fifth column. In this case, the microscope parameters computed through the metaheuristic algorithm are indicated in the first through eighth rows of Table 3. the next rows indicate the units of the variables of crack contour modeling.

In this microscope vision system, the radial distortion is determined through the coordinates of the laser line position (x*_i_*_,_*_j_*, y*_i_*_,_*_j_*), which are computed by means of Equations (20) and (21). In this way, the true coordinates are determined from distorted coordinates (x*_i_*_,_*_j_*, y*_i_*_,_*_j_*) through the expressions x*_i_*_,_*_j_*= x*_i_*_,_*_j_* + δx*_i_* and y*_i_*_,_*_j_* = y*_i_*_,_*_j_* + δy*_j_*, where (δx*_i_*, δy*_j_*) represent the distortion. Thus, a distorted line shifting is computed by the expression *S_i_*_,_*_j_* = x_1,_*_j_* − x*_i_*_,_*_j_*, while the expression *s_i_*_,_*_j_* = (x_1,_*_j_* + δx_1_) − (x*_i_*_,_*_j_* + δx*_i_*) computes the undistorted line shifting. Therefore, the distortion along the *x*-axis is calculated by the expression δx*_i_* = (x_1,_*_j_* − x*_i_*_,_*_j_*) − *s_i_*_,_*_j_* + δx_1_ = *S_i_*_,_*_j_* − *s_i_*_,j_ + δx_1_. To do this, the laser line is positioned near to the image center to obtain the initial line shifting without distortion, where δx_1_ = 0, and *s*_1_*_,j_* = *S*_1,_*_j_*. In this way, the expression *s_i_*_,_*_j_* = i*S_1_*_,j_* determines the undistorted shifting, while the distortion along the *x*-axis is calculated by the expression δx*_i_* = (x_1,_*_j_* − x*_i_*_,_*_j_*) − i*S_1,_*_j_*. Similarly, the distortion in the *y*-axis is deduced from the expressions (y*_i_*_,1_ − y*_i_*_,_*_j_*) = (y*_i_*_,1_ + δy_1_) − (y*_i_*_,_*_j_* + δy*_j_*) and *T_i_*_,_*_j_* = (y*_i_*_,1_ − y*_i_*_,_*_j_*). With these terms, the expression δy*_j_* = (y*_i_*_,1_ − y*_i_*_,_*_j_*) − j*T_i__,1_ is deduced to calculate the distortion along the *y*-axis. The results of the micro-scale modeling are described in Section 3.

## 3. Micro-Scale Crack Contour Modeling Results

**Figure 7:**
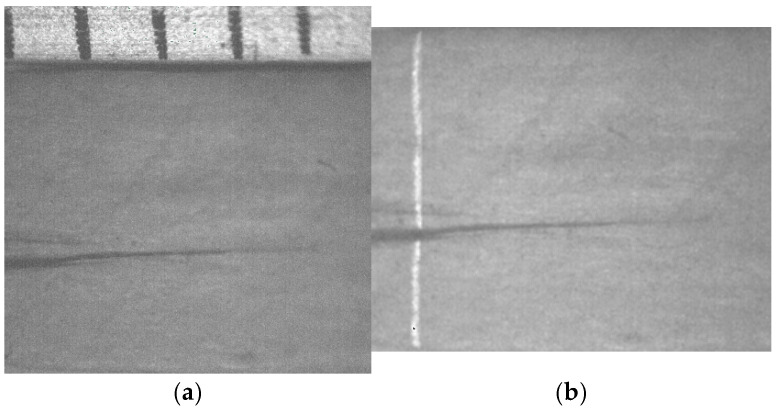
(**a**) Wood surface for performing crack contour modeling with scale in mm along the *x*-axis. (**b**) Micro-laser line projected on the wood surface to retrieve the crack region.

**Figure 8:**
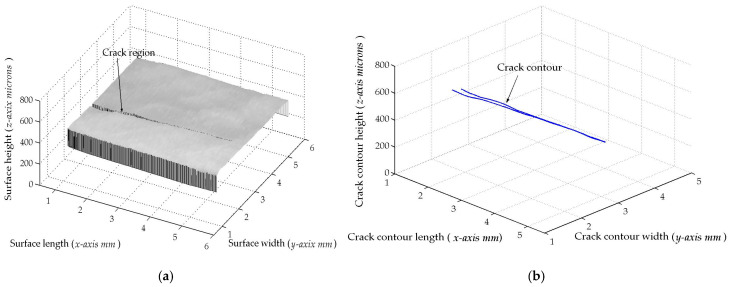
(**a**) Micro-scale wood surface recovered via micro-laser line scanning. (**b**) Crack contour constructed via crack contour model based on Bezier functions.

The micro-scale crack contour modeling is carried out by the microscope vision system shown in Figure 4a. Thus, the first micro-scale crack contour modeling is performed for the wood surface shown in Figure 7a, where the scale is indicated in millimeters along the *x*-axis. Also, the micro-laser line projected on the wood surface is shown in Figure 7b, where the broken laser line indicates the crack area along the *y*-axis. In this way, the wood surface is scanned along the *x*-axis to determine the laser line coordinates (x*_i_*_,_*_j_*, y*_i_*_,_*_j_*) by computing Equations (20) and (21). Then, x*_i_*_,_*_j_* is replaced in Equation (18) to compute the surface height *z_i_*_,_*_j_*, while y*_i,j_* is substituted in Equation (19) to calculate the surface width *y_i_*_,_*_j_*. Additionally, the slider device provides the surface length *x_i_*_,_*_j_*. Thus, two hundred and eighty-four images were processed of the wood surface shown in Figure 8a, where the *x*-axis and *y*-axis are indicated in mm, while the *z*-axis is indicated in microns. The surface-recovering accuracy is determined through the relative error [36] with the following expression:

(24) $$Er\%=\frac{100}{n\cdot m}\sum_{i=0}^{n}\sum_{j=0}^{m}\frac{\left|z_{i,j}-h_{i,j}\right|}{h_{i,j}}.$$

In this equation, *z_i_*_,_*_j_* represents the surface computed via Equation (18), *h_i_*_,_*_j_* denotes the surface measured through a contact method, and *n*·*m* is the data number. Thus, Equation (24) is computed for the wood surface illustrated in Figure 8a, resulting in a relative error of *Er*% = 1.6216%. The position of the crack contour is obtained during the surface scanning, starting from the beginning and the end of the broken laser position along the *y*-axis. In this procedure, the broken laser line is identified by summing the intensity of five adjacent pixels along the *x*-axis. Hence, if the intensity summation exceeds 680, the laser line exists. If not, the laser line is nonexistent. In this case, a crack region is identified, and the laser line position is not computed. But, the crack contour position is acquired from the last laser position along the *y*-axis. Thus, the beginning of the crack region has been obtained along the *y*-axis. Then, the procedure calculates the maximum in the subsequent rows until to it achieves an intensity summation exceeding 680. Thus, the final location of the crack region is determined in the *y*-axis.

In this way, a crack region is identified when there are three rows of broken laser lines on the *y*-axis and three broken laser lines on the *x*-axis. Based on these criteria, the coordinates (*x_i_*, *y_j_*, *z_i_*) of the broken line are acquired during the scanning of the wood surface. From these crack surface coordinates, a crack contour model is built through the metaheuristc algorithm that relies on Bezier functions as described in Section 2.2. Thus, the first step generates the search space by computing Equations (2)–(4) employing P*_r_*_+5_*_s_* = *x_r_*_+5_*_s_*, P*_r_*_+5_*_s_* = *y_r_*_+5_*_s_*, P*_r_*_+5_*_s_* = *z_r_*_+5_*_s_*. If *Z_s_*(*w_r_*) exceeds *z_r_*_+5_*_s_*, the maximum and minimum values of *w_r_*_+5_*_s_* are 1 and 0.3. But, if *Z_s_*(*w_r_*) is below *z_r_*_+5_*_s_*, the maximum and minimum values of *w_r_*_+5_*_s_* are 1.7 and 1. Also, if *Y*_s_(*v_r_*) exceeds *y_r_*_+5_*_s_*, the maximum and minimum values of w*_r_*_+5_*_s_* are 1 and 0.3. But, if *Y*_s_(*v_r_*) falls below *y_r_*_+5_*_s_*, the maximum and minimum values of w*_r_*_+5_*_s_* are 1.7 and 1. Similarly, if *X*_s_(*u_r_*) exceeds *x_r_*_+5_*_s_*, the maximum and minimum values of *w_r_*_+5_*_s_* are 1 and 0.3. But, if *X*_s_(*u_r_*) is below *x_r_*_+5_*_s_*, the maximum and minimum values of *w_r_*_+5_*_s_* are 1.7 and 1. Subsequently, four values (W_1,_*_k_*, W_2,_*_k_*, W_3,_*_k_*, W_4,_*_k_*) are randomly selected between the maximum and minimum to obtain the initial population for each weight. Next, the second step generates the current children (C_1,_*_k_*, C_2,_*_k_*, C_3,_*_k_*, C_4,_*_k_*, C_5,_*_k_*, C_6,_*_k_*) by computing Equations (11)–(16). Also, the control points P_1+5_*_s_* = (*x*_0+5_*_s_* + *x*_1+4_*_s_*)/2, P_1+5_*_s_* = (*y*_0+5_*_s_* + *y*_1+4_*_s_*)/2, P_1+5_*_s_* = (*z*_0+4_*_s_* + *z*_1+4_*_s_*)/2, P_4+5_*_s_* = (*x*_3+4_*_s_* + *x*_4+4_*_s_*)/2, P_4+5_*_s_* = (*y*_3+4_*_s_* + *y*_4+4_*_s_*)/2, P_4+5_*_s_* = (*z*_3+4_*_s_* + *z*_4+4_*_s_*)/2 are computed to provide continuity *G*^1^. Subsequently, the third step calculates the fitness by replacing [*X_s_*(*u_r_*), *Y_s_*(*v_r_*), *Z_s_*(*w_r_*)] and [*x_r_*, *y_r_*, *z_r_*] in Equation (17). Then, the fourth step determines the (*k* + 1)-generation parents by selecting the parents [W_1,_*_k_*_+1_, W_2,_*_k_*_+1_, W_3,_*_k_*_+1_, W_4,_*_k_*_+1_] from [(W_1,_*_k_*, W_2,_*_k_*), (C_1,_*_k_*, C_2,_*_k_*, C_3,_*_k_*), (W_3,_*_k_*, W_4,_*_k_*), (C_4,_*_k_*, C_5,_*_k_*, C_6,_*_k_*)]. Next, the fifth step mutates the last fit parent, introducing a new parent. Thus, if the new parent enhances the fitness, the worst parent undergoes mutation. If not, the mutation does not occur. Additionally, a new weight takes the place of a weight that is randomly selected. Thus, if the new weight improves the fitness, weight mutation takes place. In other cases, the chosen weight remains unchanged. Also, the (*k* + 1)-generation children are generated by computing Equations (11)–(16). Next, steps two through five are repeated until the optimal weights that minimize the objective function Equation (17) are obtained. Thus, a fifth Bezier curve has been obtained. This procedure is carried out to compute the Bezier curves [*X*_0_(*u*), *Y*_0_(*v*), *Z*_0_(*w*)], [*X*_1_(*u*), *Y*_1_(*v*), *Z*_1_(*w*)], [*X*_2_(*u*), *Y*_2_(*v*), *Z*_2_(*w*)], [*X*_3_(*u*), *Y*_3_(*v*), *Z*_3_(*w*)], …, [*X_M_*(*u*), *Y_M_*(*v*), *Z_M_*(*w*)] in order to generate the crack contour model. The contour curve provided by the crack contour model is shown in Figure 8b. The accuracy provided by the crack contour model is computed by the following expression:

(25) $$error\%=\frac{100}{M}\sum_{s=0}^{s=M}\sum_{r=0}^{r=5}\left[\frac{\left|Z_{s}(u_{r})-z_{r+5s}\right|}{3z_{r+5s}}+\frac{\left|Y_{s}(u_{r})-y_{r+5s}\right|}{3y_{r+5s}}+\frac{\left|X_{s}(u_{r})-x_{r+5s}\right|}{3x_{r+5s}}\right]$$

By computing Equation (25), the crack contour model produces a relative error of 0.872% for the crack contour shown in Figure 8b. Thus, the crack contour model has been constructed by the metaheuristic algorithm.

**Figure 9:**
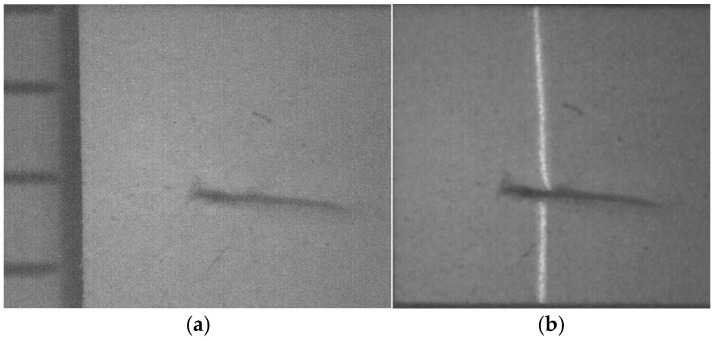
(**a**) Paper surface for performing crack contour modeling with scale in mm along the *y*-axis. (**b**) Micro-laser line projected on the paper surface to retrieve the crack region.

**Figure 10:**
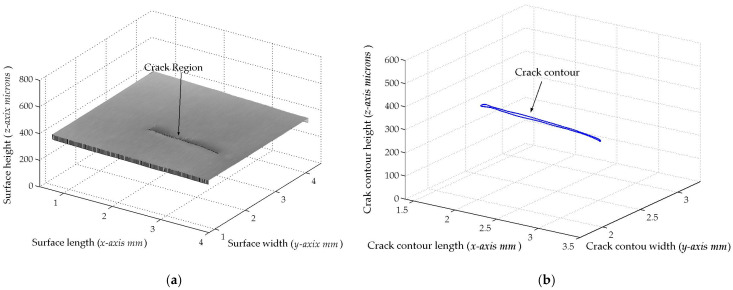
(**a**) Micro-scale paper surface recovered via micro-laser line scanning. (**b**) Crack contour constructed via crack contour modeling based on Bezier basis function.

The second micro-scale crack contour modeling is performed for the paper surface shown in Figure 9a, where the scale is indicated in millimeters along the *y*-axis. Also, the micro-laser line projected on the paper surface is illustrated in Figure 9b, where the broken laser line depicts the crack region along the *y*-axis. Thus, the paper surface is scanned along the *x*-axis to determine the surface height *z_i_*_,_*_j_* and the surface width *y_i_*_,_*_j_* by computing Equations (18) and (19) using the coordinates (x*_i_*_,_*_j_*, y*_i_*_,_*_j_*), respectively. Moreover, the slider device provides the coordinate *x_i_*_,_*_j_*. Thus, two hundred and twenty-two images were processed of the paper surface illustrated in Figure 10a, where the *x*-axis and *y*-axis are represented in millimeters, while the *z*-axis is indicated in microns. The accuracy is computed by employing the surface *z_i_*_,_*_j_* and the surface *h_i_*_,_*_j_* provided by a contact method. Thus, Equation (24) is computed for the paper surface shown in Figure 10a, resulting in a relative error of *Er*% = 1.524%. Additionally, the coordinates of the crack surface are collected during the scanning from the beginning and end of the broken laser position along the *y*-axis. In this procedure, the broken laser line is detected when the sum intensity of five adjacent pixels along the *x*-axis falls below 680. When the laser line does not exist, the crack contour position is obtained from the last laser position along the *y*-axis. Thus, the beginning of the crack region is obtained along the *y*-axis. Next, the maximum is computed in the subsequent rows until achieving an intensity summation exceeding 680 to determine the end position in the *y*-axis. Thus, a crack region is detected when there are three rows of the broken laser line in the *y*-axis and three broken laser lines in the *x*-axis. In this way, the broken line coordinates are computed during the paper surface scanning. Then, the crack contour model is built using the metaheuristc algorithm through the Bezier functions. Thus, the first step computes Equations (2)–(4) via P*_r_*_+5_*_s_* = *x_r_*_+5_*_s_*, P*_r_*_+5_*_s_* = *y_r_*_+5_*_s_*, P*_r_*_+5_*_s_* = *z_r_*_+5_*_s_* to establish the initial population. If *Z_s_*(*w_r_*) is over *z_r_*_+5_*_s_*, the maximum and minimum values of *w_r_*_+5_*_s_* are 1 and 0.3. If not, the maximum and minimum values of *w_r_*_+5_*_s_* are 1.7 and 1. Also, if *Y*_s_(*v_r_*) exceeds *y_r_*_+5_*_s_*, the maximum and minimum values of w*_r_*_+5_*_s_* are 1 and 0.3. Otherwise, the maximum and minimum are 1.7 and 1. Additionally, if *X*_s_(*u_r_*) exceeds *x_r_*_+5_*_s_*, the maximum and minimum values of *w_r_*_+5_*_s_* are 1 and 0.3. If not, the maximum and minimum are 1.7 and 1. Then, four values (W_1,_*_k_*, W_2,_*_k_*, W_3,_*_k_*, W_4,_*_k_*) are randomly selected between the maximum and minimum to determine the initial population for each weight. Then, the second step computes Equations (11)–(16) to obtain the current children (C_1,_*_k_*, C_2,_*_k_*, C_3,_*_k_*, C_4,_*_k_*, C_5,_*_k_*, C_6,_*_k_*). Also, P_1+5_*_s_* = (*x*_0+5_*_s_* + *x*_1+4_*_s_*)/2, P_1+5_*_s_* = (*y*_0+5_*_s_* + *y*_1+4_*_s_*)/2, P_1+5_*_s_* = (*z*_0+4_*_s_* + *z*_1+4_*_s_*)/2, P_4+5_*_s_* = (*x*_3+4_*_s_* + *x*_4+4_*_s_*)/2, P_4+5_*_s_* = (*y*_3+4_*_s_* + *y*_4+4_*_s_*)/2, P_4+5_*_s_* = (*z*_3+4_*_s_* + *z*_4+4_*_s_*)/2 are computed to provide continuity *G*^1^.

Then, the third step computes the fitness Equation (17) based on [*X_s_*(*u_r_*), *Y_s_*(*v_r_*), *Z_s_*(*w_r_*)] and [*x_r_*, *y_r_*, *z_r_*]. Next, the fourth step selects the (*k* + 1)-generation parents [W_1,_*_k_*_+1_, W_2,_*_k_*_+1_, W_3,_*_k_*_+1_, W_4,_*_k_*_+1_] from [(W_1,_*_k_*, W_2,_*_k_*), (C_1,_*_k_*, C_2,_*_k_*, C_3,_*_k_*), (W_3,_*_k_*, W_4,_*_k_*), (C_4,_*_k_*, C_5,_*_k_*, C_6,_*_k_*)], respectively. Then, the fifth step performs a mutation by replacing the least fit parent with a new one. Also, a new weight replaces a weight that is selected at random. Then, Equations (11)–(16) are computed to obtain the (*k*+1)-generation children. Subsequently, steps two through five are repeated until the optimal weights that minimize the objective function Equation (17) are obtained. This procedure is carried out to compute the optimal Bezier curves [*X*_0_(*u*), *Y*_0_(*v*), *Z*_0_(*w*)], [*X*_1_(*u*), *Y*_1_(*v*), *Z*_1_(*w*)], [*X*_2_(*u*), *Y*_2_(*v*), *Z*_2_(*w*)], [*X*_3_(*u*), *Y*_3_(*v*), *Z*_3_(*w*)], …, [*X_M_*(*u*), *Y_M_*(*v*), *Z_M_*(*w*)] that represent the crack contour model. From this crack contour model, the crack contour shown in Figure 10b is obtained. The accuracy provided by the crack contour model is computed via Equation (25), and the result is a relative error of 0.9612%. Thus, the micro-scale crack contour model has been built using the metaheuristic algorithm and micro-laser line scanning.

**Figure 11:**
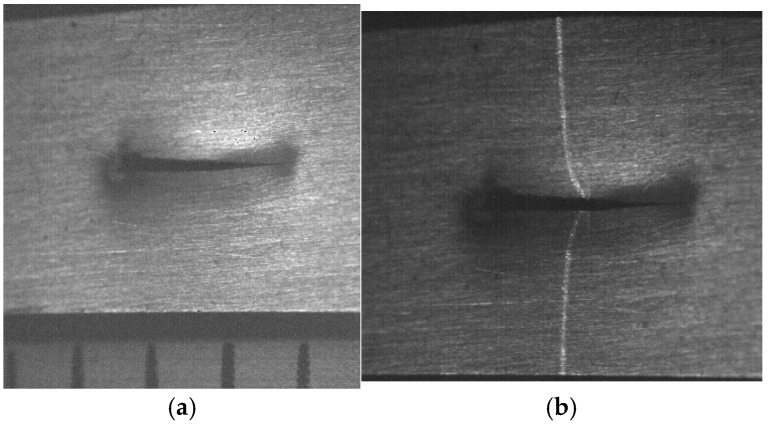
(**a**) Metallic surface for performing crack contour modeling with scale in mm on *x*-axis. (**b**) Micro-laser line projected on the metallic surface to retrieve the crack region.

**Figure 12:**
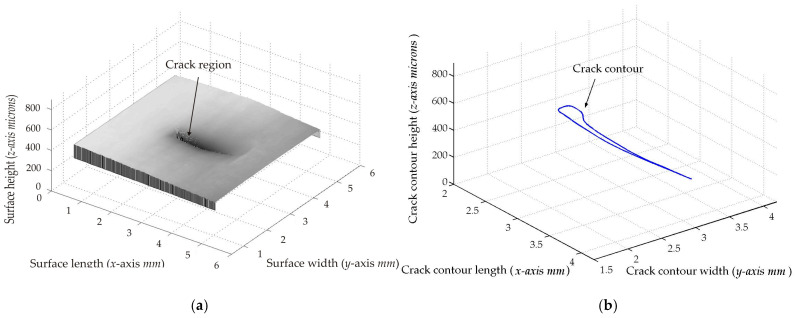
(**a**) Micro-scale metallic surface recovered via micro-laser line scanning. (**b**) Crack contour constructed via crack contour modeling.

The third micro-scale crack contour modeling is performed for the metallic surface illustrated in Figure 11a, where the scale is indicated in millimeters along the *x*-axis. Figure 11b illustrates the broken laser line within the crack region of the metallic surface along the *y*-axis. Thus, the metallic surface is scanned at the *x*-axis to retrieve the coordinates (x*_i_*_,_*_j_*, y*_i_*_,_*_j_*). At the same time, the surface height *z_i_*_,_*_j_* and surface width *y_i_*_,_*_j_* are retrieved by computing Equations (18) and (19) by means of the coordinates (x*_i_*_,_*_j_*, y*_i_*_,_*_j_*). Additionally, the slider device provides the coordinate *x_i_*_,_*_j_*. Thus, two hundred and seventy-four images were processed of the metallic surface shown in Figure 12a, where the *x*-axis and *y*-axis are indicated in millimeters, while the *z*-axis is indicated in microns. The accuracy is computed via relative error using the coordinate *z_i_*_,_*_j_* and the surface *h_i_*_,_*_j_* measured by a contact method. Thus, Equation (24) is computed for the metallic surface shown in Figure 12a, resulting in a relative error of 1.436%. Additionally, the coordinates of the crack surface are computed during the scanning based on the position of the broken laser line along the *y*-axis. Thus, the broken laser line is detected when the sum of five adjacent pixels along the *x*-axis falls below 680. Also, the crack contour position is obtained from the last laser position on the *y*-axis to establish the beginning of the crack region. Then, the summation is computed in the next rows to find a value greater than 680, which determines the end position of the crack region on the *y*-axis. Also, a crack region is detected when there are three rows of broken laser line on the *y*-axis and three broken laser lines on the *x*-axis. In this way, the broken line coordinates are obtained during the scanning. Then, the crack contour model is constructed by the metaheuristc algorithm via Bezier functions. Thus, the first step computes Equations (2)–(4) to determine the initial population. If *Z_s_*(*w_r_*) exceeds *z_r_*_+5_*_s_*, the maximum and minimum values of *w_r_*_+5_*_s_* are 1 and 0.3. Otherwise, the maximum and minimum values of *w_r_*_+5_*_s_* are 1.7 and 1. Also, if *Y*_s_(*v_r_*) exceeds *y_r_*_+5_*_s_*, the maximum and minimum values of w*_r_*_+5_*_s_* are 1 and 0.3. If not, the maximum and minimum are 1.7 and 1. Additionally, if *X*_s_(*u_r_*) exceeds *x_r_*_+5_*_s_*, the maximum and minimum values of *w_r_*_+5_*_s_* are 1 and 0.3. If not, the maximum and minimum are 1.7 and 1. Then, four values (W_1,_*_k_*, W_2,_*_k_*, W_3,_*_k_*, W_4,_*_k_*) are randomly selected from the search space to obtain the initial population for each weight. Then, the second step computes Equations (11)–(16) to generate the current children (C_1,_*_k_*, C_2,_*_k_*, C_3,_*_k_*, C_4,_*_k_*, C_5,_*_k_*, C_6,_*_k_*). Also, P_1+5_*_s_* = (*x*_0+5_*_s_* + *x*_1+4_*_s_*)/2, P_1+5_*_s_* = (*y*_0+5_*_s_* + *y*_1+4_*_s_*)/2, P_1+5_*_s_* = (*z*_0+4_*_s_* + *z*_1+4_*_s_*)/2, P_4+5_*_s_* = (*x*_3+4_*_s_* + *x*_4+4_*_s_*)/2, P_4+5_*_s_* = (*y*_3+4_*_s_* + *y*_4+4_*_s_*)/2, and P_4+5_*_s_* = (*z*_3+4_*_s_* + *z*_4+4_*_s_*)/2 are computed to provide continuity *G*^1^.

Next, the third step computes the fitness Equation (17) employing [*X_s_*(*u_r_*), *Y_s_*(*v_r_*), *Z_s_*(*w_r_*)] and [*x_r_*, *y_r_*, *z_r_*]. Then, the fourth step selects the (*k* + 1)-generation parents [W_1,_*_k_*_+1_, W_2,_*_k_*_+1_, W_3,_*_k_*_+1_, W_4,_*_k_*_+1_] from [(W_1,_*_k_*, W_2,_*_k_*), (C_1,_*_k_*, C_2,_*_k_*, C_3,_*_k_*), (W_3,_*_k_*, W_4,_*_k_*), (C_4,_*_k_*, C_5,_*_k_*, C_6,_*_k_*)], respectively. Next, the fifth step performs the mutation of the worst parent and the mutation of a weight. Also, Equations (11)–(16) are computed to obtain the (*k* + 1)-generation children. Then, steps two through five are repeated to obtain the optimal weights that minimize Equation (17). This procedure is carried out to compute the optimal Bezier curves [*X*_0_(*u*), *Y*_0_(*v*), *Z*_0_(*w*)], [*X*_1_(*u*), *Y*_1_(*v*), *Z*_1_(*w*)], [*X*_2_(*u*), *Y*_2_(*v*), *Z*_2_(*w*)], [*X*_3_(*u*), *Y*_3_(*v*), *Z*_3_(*w*)], …, [*X_M_*(*u*), *Y_M_*(*v*), *Z_M_*(*w*)] that represent the crack contour model. Thus, the crack contour illustrated in Figure 12b is obtained from the crack contour model. The accuracy of the crack contour model is computed via Equation (25), obtaining a relative error of 0.8964%. Thus, the micro-scale crack contour model has been constructed by the metaheuristic algorithm and micro-laser line scanning.

**Figure 13:**
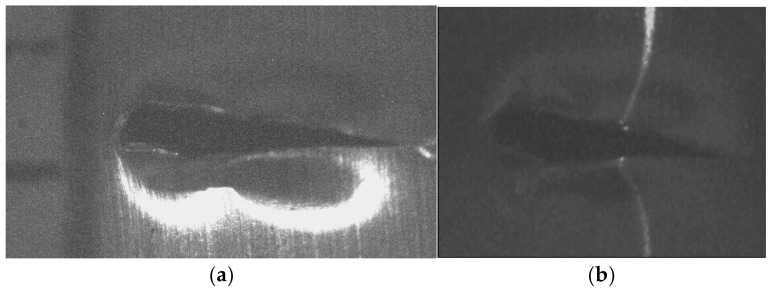
(**a**) Non-planar surface with high reflectivity for performing crack contour modeling with scale in mm on the *y*-axis. (**b**) Micro-laser line projected on the non-planar surface to retrieve the crack region.

**Figure 14:**
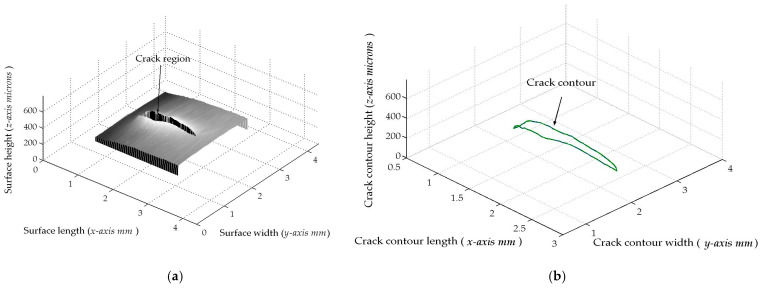
(**a**) Micro-scale non-planar surface recovered via micro-laser line scanning. (**b**) Crack contour constructed via crack contour model based on Bezier functions.

**Figure 15:**
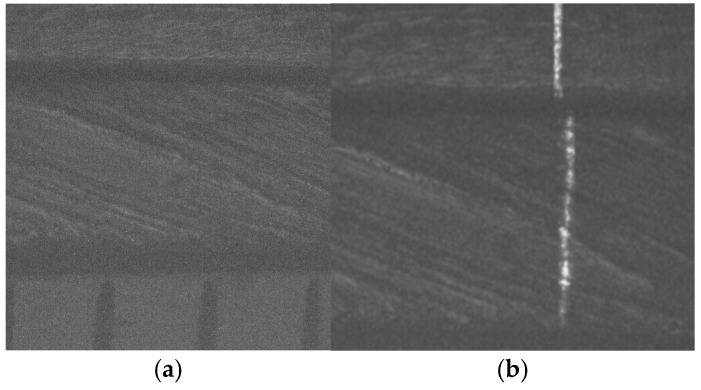
(**a**) Texture surface for performing crack contour modeling with scale in mm on *x*-axis. (**b**) Micro-laser line projected on the texture surface to retrieve crack surface.

The fourth micro-scale crack contour modeling is performed for a non-planar crack surface with high reflectivity, which is shown in Figure 13a, where the scale is represented in millimeters on the *y*-axis. Additionally, the high reflectivity is illustrated by the brightness produced on the surface. This brightness produces saturation intensity in the image. The saturation intensity is detected when every pixel in a region exceeds 255. To avoid high brightness, the system reduces the light fed into the CCD array. In this way, the laser line is observed without interference with the surface brightness as shown in Figure 13b. Also, the broken laser line indicates the crack region along the *y*-axis. Thus, the non-planar surface is scanned along the *x*-axis to determine the coordinates (x*_i_*_,_*_j_*, y*_i_*_,_*_j_*) by computing Equations (20) and (21). These equations provide a smooth curve from the pixel intensity, reducing noise in the laser line’s pixels and inaccuracies. This criterion is elucidated by the Bezier curve shown in Figure 2. Thus, the surface height *z_i_*_,_*_j_* and the surface width *y_i_*_,_*_j_* are determined by computing Equations (18) and (19) using the coordinates (x*_i_*_,_*_j_*, y*_i_*_,_*_j_*), respectively. Additionally, the coordinate *x_i_*_,_*_j_* is provided by the slider device. Thus, two hundred and forty-six images were processed of the non-planar surface shown in Figure 14a, where the *x*-axis and *y*-axis are indicated in millimeters, while the *z*-axis is represented in microns. The accuracy is computed by employing the surface *z_i_*_,_*_j_* and the surface *h_i_*_,_*_j_* provided by a contact method. Thus, Equation (24) is computed for the non-planar crack surface shown in Figure 15a, and the result is a relative error of *Er*% = 1.672%. Additionally, the coordinates of the crack surface are collected from the beginning and end of the broken laser position along the *y*-axis. The broken laser line is detected when the intensity sum of five adjacent pixels along the *x*-axis falls below 680. In the absence of the laser line, the crack contour position is obtained from the last laser position in the *y*-axis. Next, the maximum is computed in the following rows to find an intensity sum over 680 to determine the end position on the *y*-axis. Thus, a crack region is detected when there are three rows of the broken laser line along the *y*-axis and three broken laser lines along the *x*-axis. In this way, the broken line coordinates are computed during the scanning of the non-planar surface. Then, the metaheuristc algorithm generates the crack contour model through the Bezier functions. Thus, the first step calculates Equations (2)–(4) via P*_r_*_+5_*_s_* = *x_r_*_+5_*_s_*, P*_r_*_+5_*_s_* = *y_r_*_+5_*_s_*, P*_r_*_+5_*_s_* = *z_r_*_+5_*_s_* to determine the initial population. If Z*_s_*(*w_r_*) exceeds *z_r_*_+5_*_s_*, the maximum and minimum values of *w_r_*_+5_*_s_* values are 1 and 0.3. If not, the maximum and minimum values of *w_r_*_+5_*_s_* are 1.7 and 1. Also, if *Y*_s_(*v_r_*) exceeds *y_r_*_+5_*_s_*, the maximum and minimum values of w*_r_*_+5_*_s_* are 1 and 0.3. Otherwise, the maximum and minimum are 1.7 and 1. Additionally, if *X*_s_(*u_r_*) exceeds *x_r_*_+5_*_s_*, the maximum and minimum values of *w_r_*_+5_*_s_* are 1 and 0.3. If not, the maximum and minimum are 1.7 and 1. Then, four values (W_1,_*_k_*, W_2,_*_k_*, W_3,_*_k_*, W_4,_*_k_*) are randomly selected within the range of the maximum and minimum to determine the initial population for every weight. Next, the second step computes Equations (11)–(16) to obtain the current children (C_1,_*_k_*, C_2,_*_k_*, C_3,_*_k_*, C_4,_*_k_*, C_5,_*_k_*, C_6,_*_k_*). Also, P_1+5_*_s_* = (*x*_0+5_*_s_* + *x*_1+4_*_s_*)/2, P_1+5_*_s_* = (*y*_0+5_*_s_* + *y*_1+4_*_s_*)/2, P_1+5_*_s_* = (*z*_0+4_*_s_* + *z*_1+4_*_s_*)/2, P_4+5_*_s_* = (*x*_3+4_*_s_* + *x*_4+4_*_s_*)/2, P_4+5_*_s_* = (*y*_3+4_*_s_* + *y*_4+4_*_s_*)/2, and P_4+5_*_s_* = (*z*_3+4_*_s_* + *z*_4+4_*_s_*)/2 are computed to provide continuity *G*^1^.

Then, the third step calculates the fitness Equation (17) employing [*X_s_*(*u_r_*), *Y_s_*(*v_r_*), *Z_s_*(*w_r_*)] and [*x_r_*, *y_r_*, *z_r_*]. Next, the fourth step selects the (*k* + 1)-generation parents [W_1,_*_k_*_+1_, W_2,_*_k_*_+1_, W_3,_*_k_*_+1_, W_4,_*_k_*_+1_] from [(W_1,_*_k_*, W_2,_*_k_*), (C_1,_*_k_*, C_2,_*_k_*, C_3,_*_k_*), (W_3,_*_k_*, W_4,_*_k_*), (C_4,_*_k_*, C_5,_*_k_*, C_6,_*_k_*)], respectively. Then, the fifth step performs a mutation by replacing the weakest parent with a new one. Also, a new weight replaces a weight that is randomly selected. Then, Equations (11)–(16) are computed to obtain the (*k* + 1)-generation children. Subsequently, the process from steps two through five is repeated to obtain the optimal weights that minimize the objective function Equation (17). This procedure is performed to determine the Bezier curves [*X*_0_(*u*), *Y*_0_(*v*), *Z*_0_(*w*)], [*X*_1_(*u*), *Y*_1_(*v*), *Z*_1_(*w*)], [*X*_2_(*u*), *Y*_2_(*v*), *Z*_2_(*w*)], [*X*_3_(*u*), *Y*_3_(*v*), *Z*_3_(*w*)], …, [*X_M_*(*u*), *Y_M_*(*v*), *Z_M_*(*w*)] that represent the crack contour model. From this crack contour model, the crack contour illustrated in Figure 14b is obtained. The accuracy obtained from the crack contour model is calculated via Equation (25), yielding a relative error of 0.9217%. Thus, the micro-scale crack contour model has been built for the non-planar crack surface using the metaheuristic algorithm.

**Figure 16:**
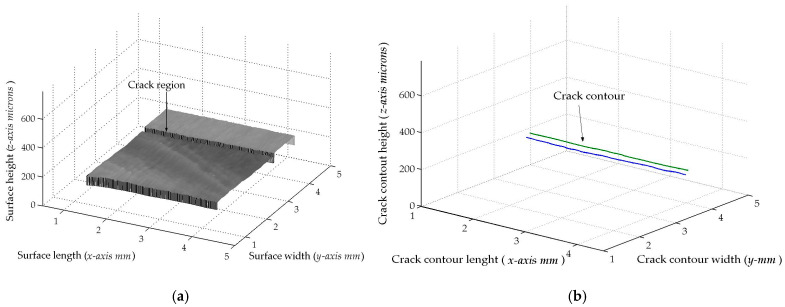
(**a**) Micro-scale texture surface recovered via micro-laser line scanning. (**b**) Crack contour constructed via crack contour modeling based on Bezier basis function.

The fifth micro-scale crack contour modeling is performed for the textured surface shown in Figure 15a, where the scale is indicated in millimeters along the *x*-axis. Thus, the micro-laser line is projected on the texture surface as shown in Figure 15b. This image elucidates the effectiveness of the micro-laser line width in avoiding interference with the texture surface. Figure 15b illustrates the laser line displacement on the *x*-axis due to the surface variation along the *z*-axis. The sensitivity of the laser line width is deduced based on the line shifting along the *x*-axis due to the surface variation along the *z*-axis. Thus, the minimum surface *z_i_*_,_*_j_* that produces the minimum line shifting (x*_i_*_,_*_j_*,–x*_c_*) = 1 pixel is determined based on the surface point *O*. In this way, Equation (18) is computed by substituting the values of the vision parameters (*x_c_*, *y_c_*, *η*, *θ*, *d*_1_, *F*_1_, *d*_2_, *F*_2_) and (x*_i_*_,_*_j_*,–x*_c_*) = 1 to obtain *z_i_*_,_*_j_*, which produces a shifting of 1 pixel along the *x*-axis. The values of the vision parameters substituted in Equation (18) are described in Section 2.4. By computing Equation (19), the result is a surface *z_i_*_,_*_j_* = 4.7 microns from the surface point *O*. Thus, the laser line width is allowed to determine surface variations of 4.7 microns along the *z*-axis. In this way, the laser line width is allowed to make displacements along the *x*-axis due to the micro-scale texture variation over 4.7 microns. This criterion is elucidated in the texture surface shown in Figure 15b, where the laser line is displaced along the *x*-axis due to the texture variation. Thus, the line displacements provide the laser line location to determine the micro-scale texture surface. Also, the broken laser line indicates the crack region along the *y*-axis. In this way, the texture surface is scanned along the *x*-axis to determine the coordinates (x*_i_*_,_*_j_*, y*_i_*_,_*_j_*) by computing Equations (20) and (21). These equations provide a smooth curve from the pixel intensity, eliminating noise in the pixels of the laser line as shown in Figure 2. During the scanning, the surface height *z_i_*_,_*_j_* and the surface width *y_i_*_,_*_j_* are calculated by computing Equations (18) and (19) employing the coordinates (x*_i_*_,_*_j_*, y*_i_*_,_*_j_*), respectively. Additionally, the slider device provides the coordinate *x_i_*_,_*_j_*. Thus, two hundred and ninety-two images were processed of the texture surface shown in Figure 16a, where the *x*-axis and *y*-axis are indicated in millimeters, while the *z*-axis is indicated in microns. The accuracy is determined by using the surface *z_i_*_,_*_j_* and the surface *h_i_*_,_*_j_* measured by a contact method. Thus, Equation (24) is calculated for the texture surface illustrated in Figure 16a, and the result is a relative error of *Er*% = 1.852%. Also, the crack surface coordinates are obtained during the scanning from the beginning and the end of the broken laser along the *y*-axis. The broken laser line is identified when the sum of the intensities of five adjacent pixels along the *x*-axis falls below 680. In this case, the position of the crack contour is acquired from the last laser position in the *y*-axis. Next, the maximum is computed in the following rows to find an intensity sum over 680 to determine the end position of the crack along the *y*-axis. Thus, a crack region is detected when there are three rows of the broken laser line pn the *y*-axis and three broken laser lines on the *x*-axis. In this way, the broken line coordinates are retrieved during the scanning of the texture surface. Then, the metaheuristc algorithm performs the crack contour model through the Bezier functions.

Thus, the first step computes Equations (2)–(4) via P*_r_*_+5_*_s_* = *x_r_*_+5_*_s_*, P*_r_*_+5_*_s_* = *y_r_*_+5_*_s_*, P*_r_*_+5_*_s_* = *z_r_*_+5_*_s_* to obtain the initial population. If *Z_s_*(*w_r_*) is greater than *z_r_*_+5_*_s_*, the maximum and minimum values of *w_r_*_+5_*_s_* are 1 and 0.3. If not, the maximum and minimum values of *w_r_*_+5_*_s_* are 1.7 and 1. Also, if *Y*_s_(*v_r_*) exceeds *y_r_*_+5_*_s_*, the maximum and minimum values of w*_r_*_+5_*_s_* are 1 and 0.3. Otherwise, the maximum and minimum are 1.7 and 1. Additionally, if *X*_s_(*u_r_*) exceeds *x_r_*_+5_*_s_*, the maximum and minimum values of *w_r_*_+5_*_s_* are 1 and 0.3. If not, the maximum and minimum are 1.7 and 1. Then, four values (W_1,_*_k_*, W_2,_*_k_*, W_3,_*_k_*, W_4,_*_k_*) are randomly selected from the maximum and minimum to establish the initial population for every weight. Then, the second step computes Equations (11)–(16) to generate the current children (C_1,_*_k_*, C_2,_*_k_*, C_3,_*_k_*, C_4,_*_k_*, C_5,_*_k_*, C_6,_*_k_*). Also, the control points P_1+5_*_s_* = (*x*_0+5_*_s_* + *x*_1+4_*_s_*)/2, P_1+5_*_s_* = (*y*_0+5_*_s_* + *y*_1+4_*_s_*)/2, P_1+5_*_s_* = (*z*_0+4_*_s_* + *z*_1+4_*_s_*)/2, P_4+5_*_s_* = (*x*_3+4_*_s_* + *x*_4+4_*_s_*)/2, P_4+5_*_s_* = (*y*_3+4_*_s_* + *y*_4+4_*_s_*)/2, P_4+5_*_s_* = (*z*_3+4_*_s_* + *z*_4+4_*_s_*)/2 are calculated to provide continuity *G*^1^.

Then, the third step evaluates the fitness Equation (17) employing [*X_s_*(*u_r_*), *Y_s_*(*v_r_*), *Z_s_*(*w_r_*)] and [*x_r_*, *y_r_*, *z_r_*]. Next, the fourth step chooses the (*k* + 1)-generation parents [W_1,_*_k_*_+1_, W_2,_*_k_*_+1_, W_3,_*_k_*_+1_, W_4,_*_k_*_+1_] from [(W_1,_*_k_*, W_2,_*_k_*), (C_1,_*_k_*, C_2,_*_k_*, C_3,_*_k_*), (W_3,_*_k_*, W_4,_*_k_*), (C_4,_*_k_*, C_5,_*_k_*, C_6,_*_k_*)], respectively. Then, the fifth step mutates the weakest parent with a new one. Also, a new weight replaces a weight that is randomly selected. Then, Equations (11)–(16) are computed to obtain the (*k* + 1)-generation children. Subsequently, the process from steps two through five is repeated to obtain the optimal weights that minimize the objective function Equation (17). This procedure is performed to determine the Bezier curves [*X*_0_(*u*), *Y*_0_(*v*), *Z*_0_(*w*)], [*X*_1_(*u*), *Y*_1_(*v*), *Z*_1_(*w*)], [*X*_2_(*u*), *Y*_2_(*v*), *Z*_2_(*w*)], …, [*X_M_*(*u*), *Y_M_*(*v*), *Z_M_*(*w*)], which represent the crack contour model of the crack contour shown in Figure 16b. The accuracy obtained from the crack contour model is calculated via Equation (25), yielding a relative error of 0.945%. Thus, the micro-scale crack contour model has been built for the textured crack surface using the metaheuristic algorithm.

**Figure 17:**
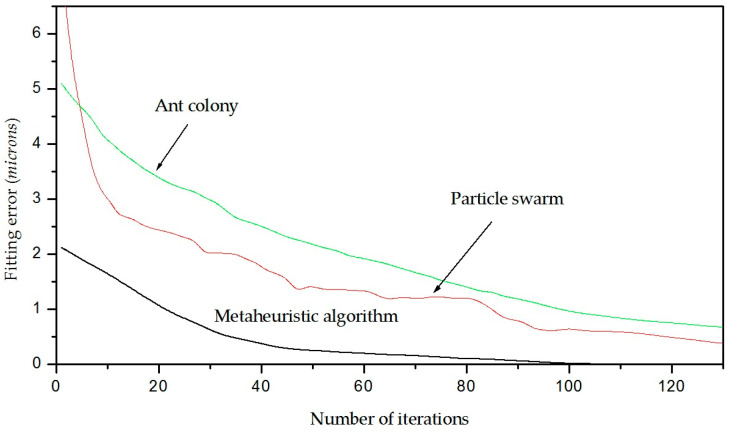
Evolution of the error of metaheuristic algorithm based on the number of iterations.

The capability of the metaheuristic algorithm is established based on the fitting accuracy of the crack contour model to represent the crack topography [37]. This capability includes model fitting accuracy and algorithm efficiency. To elucidate these criteria, the fitting algorithm accuracy and the number of iterations are described as follows. In this way, the error of the algorithm is determined during the iterations to construct the crack contour model. This error is determined by computing the average error of the crack contour model of the wood surface, paper surface, metallic surface, non-planar surface with a high reflectance, and texture surface. From these crack contour models, the average error in the first iteration is 2.261 microns. Then, the error decreases in the next iterations, obtaining an average error of 0.000651 in iteration 108. The behavior of the error of the metaheuristic algorithm in these crack contour models is depicted in Figure 17, where the black line represents the error of the metaheuristic algorithm during the iterations.

Also, the errors of algorithms such as particle swarm and ant colony are shown as a reference for accuracy. Based on these results, the metaheuristic algorithm provides a good fitting accuracy and efficiency. Additionally, the number of operations for one Bezier curve [*X_s_*(*u_r_*), *Y_s_*(*v_r_*), *Z_s_*(*w_r_*)] in each iteration is established to determine the efficiency. Based on Equations (2)–(4), the number of operations to compute the expressions [*X_s_*(*u_r_*), *Y_s_*(*v_r_*), *Z_s_*(*w_r_*)] is 153, including sums and multiplications.

The computer employed to perform the micro-scale crack contour modeling is a PC at 2.4 GHz of velocity. In this computer, 68 images are captured per second by means of the CCD camera. Also, the computer moves the slider device by means of a control software to perform the micro-laser line scanning via optical microscope. Thus, a laser line image is processed in 0.0046 s to determine the topography of a transverse section. The crack contour model for the wood surface was computed in 33.21 s. This time includes the surface scanning and the crack contour modeling. In the same way, the crack contour model for the paper surface was obtained in 31.21 s, the crack contour model for the metallic surface was determined in 33.87 s, the crack contour model for the non-planar surface of high reflectance was computed in 36.35 s, and the crack contour model for the texture surface was obtained in 37.12 s.

## 4. Discussion

Typically, the viability of crack modeling is established based on the accuracy in detecting and characterizing crack contour regions [38]. In this way, the contribution of the proposed metaheuristic algorithm is established based on the accuracy of the crack contour models in representing crack regions. This statement includes the accuracy of the crack contour models and crack surface detection. In these matters, the metaheuristic algorithm generates crack contour models that accurately fit a Bezier curve toward the crack topography in efficient form. The accuracy of the crack contour models is determined by means of the relative error of Equation (25), which represents the quality gap of the algorithm. In this way, the crack contour fitting is accomplished by moving the Bezier curves toward the crack topography by means of the control points (P, P, P). Thus, the metaheuristic algorithm generates crack contour models that represent crack surface contours with a relative error smaller than 1.0*%*. Additionally, crack surface detection through the broken micro-laser line provides high efficiency in detecting crack regions. This is because the micro-laser line is broken in a crack region. Thus, the broken laser line determines the real crack region based on the laser line coordinates. Therefore, the crack contour model accurately represents the real crack surface contour. This procedure improves the accuracy of the inspection of crack regions to determine surface quality. Additionally, the metaheuristic algorithm’s efficiency is established based on the algorithm structure and solution quality. In this case, the metaheuristic algorithm provides a suitable structure for adjusting the crack contour model to the crack topography. This is because the metaheuristic algorithm moves the Bezier functions toward the crack surface coordinates, adjusting the crack contour model. Moreover, the research space is deduced from the crack topography to optimize the weights of the crack contour model. In this way, a good fit is achieved after the first generation. Thus, a moderated number of iterations optimizes the crack contour model, where each iteration performs 153 operations to compute the expressions [*X_s_*(*u_r_*), *Y_s_*(*v_r_*), *Z_s_*(*w_r_*)] of the crack contour model. Thus, the metaheuristic algorithm achieves a moderated running time. Moreover, the micro-laser line scanning accuracy has an influence on the accuracy of the crack contour model. This is because micro-laser line projection provides good accuracy in retrieving the real crack topography. Based on these statements, the proposed metaheuristic algorithm improves the crack contour modeling of the traditional algorithms. This is because the traditional algorithms based on image intensity distribution characterize the crack models with a relative error over 8.0% [39,40]. Additionally, algorithms of artificial intelligence have been implemented to perform characterization of crack regions [41]. These algorithms characterize crack regions through the traditional structure [42] in which the research space is not deduced from the crack topography. Instead, the metaheuristic algorithm determines the solution space from the crack topography. Therefore, the metaheuristic algorithm optimizes the crack contour model based on the crack surface coordinates, where the control points move the crack contour toward the crack topography. In this way, the structure provides a low error after the first iterations. This is one difference from the traditional algorithms, which generate the initial population in random form [43]. Also, the proposed algorithm searches inside and outside parents to find the optimal weights. This procedure avoids the elimination of potential candidates to find the optimal solution. To elucidate the contribution of the crack contour modeling, the accuracy of the traditional crack characterization methods is as follows. Typically, crack modeling and detection are performed with a relative error over 8% by means of the traditional optical microscope systems [44], where the crack surface data are determined through gray-scale microscope images. Therefore, the traditional optical microscope techniques characterize the crack regions through the image intensity distribution [45]. Therefore, these microscope imaging systems do not characterize the crack region in accurate form. This is because the intensity profile does not accurately characterize the crack topography. Instead, the micro-laser line accurately depicts the crack topography via microscope images. Therefore, the crack is always detected through the broken micro-laser line. This statement has been elucidated through the crack surface detection shown in Section 3, where the crack contour models were fitted with a relative error of 0.945%. On the other hand, the characterization and detection accuracy of the traditional algorithms is computed by the expression Accuracy = (TP + TN)/(TP + FP + TN + FN), where TP indicates the true positives, FP represents the false positives, TN depicts the true negatives, and FN represents the false negatives. For instance, crack characterization via Gaussian filtering provides a relative error over 8% for crack modeling and detection [46]. Also, crack characterization via homomorphic filtering performs crack contouring and detection with a relative error over 8% for [47]. In the same way, Laplacian–Gaussian filtering performs crack contouring and detection with a relative error over 8% [48]. Moreover, crack modeling via segmentation performs crack contouring and detection with a relative error over 8% [49]. Also, skeletonization based on segmentation performs crack contour characterization with a relative error over 8% [50]. Furthermore, frequency domain methods yield crack contouring with a relative error over 8% [51]. In this field, the Fourier transform method carries out crack modeling with an accuracy of 86% [52]. On the other hand, deep learning methods realize crack contouring with a relative error over 8% [53]. In this field, convolutional neural networks perform crack modeling with an accuracy of 90% [54]. Also, crack contour edge detection via convolutional neural networks achieve an accuracy of 92% [55]. But, residual neural networks perform crack contour modeling with a relative error over 8% [56]. Also, crack contour edge detection via YoloV8 is achieved with an error of 5% [57]. Based on these statements, it is established that the proposed metaheuristic algorithm and micro-laser projection enhance the accuracy of traditional crack characterization and detection.

Additionally, the proposed metaheuristic algorithm was examined based on traditional metaheuristic algorithms such as particle swarm and ant colony. To accomplish this, the accuracy of crack characterization and detection of traditional metaheuristic algorithms was examined. The particle swarm algorithm achieves crack characterization and detection with a relative error over 8% [58]. Moreover, ant colony optimization performs crack contouring and detection with a relative error over 8% [59]. Also, the metaheuristic algorithm structure was examined based on the particle swarm structure, which is very useful in the contour curves optimization [60]. The particle swarm method computes the velocity and position of a particle to determine the population of each generation. Thus, the expression *V_i_*(*t* + 1) = *wV_i_*(*t*) + *αR*_1_[*P^b^_g_*(*t*) − *P_i_*(*t*)] + *βR*_2_[*P^b^_i_*(*t*) − *P_i_*(*t*)] computes the velocity of the particle, where *w* is an inertia weight, *t* is the number of iterations, (*α*, *β*) represent the learning factors, and (*R*_1_, *R*_2_) are selected in random form in the interval from 0 to 1. The position of the particle is computed by the expression *P_i_*(*t* + 1) = *P_i_*(*t*) + *V_i_*(*t +* 1). Therefore, the particle swarm computes five parameters to create the population in each *k*-generation. But, these additional parameters are not related to the crack topography. Based on these statements, the metaheuristic algorithm and particle swarm structure can be examined to build crack contour models. To do this, the particle swarm can be utilized based on the contour models of the wood surface, paper surface, metallic surface, surface with high reflectance, and texture surface. Thus, the first examination involves the initial population, where the particle swarm randomly selects the range of the particles. Instead, the metaheuristic algorithm determines the range of every weight by computing Equations (2)–(4) based on the surface topography. Thus, the range of each particle is defined as [−2, 2] to compute the initial particles. But, the first step of the metaheuristic algorithm determines the range of every weight based on the initial Bezier curve and crack surface as described in Section 2.2. From this stage, the metaheuristic algorithm produces smaller errors after the first iteration. The second examination is the number of tested particles, where the particle swarm initially defines the number of particles. This procedure leads to falls in a local minimum. Instead, the metaheuristic algorithm begins with four parents, but a new parent and a new weight are tested in each new generation to avoid a local minimum. This procedure is performed by the mutation described in Section 2.2. Thus, 40 particles are defined to perform the particle swarm, and the metaheuristic algorithm tests 240 potential candidates. The third assessment is the population for the next iteration, where the particle swarm computes *V_i_*(*t* + 1) and *P_i_*(*t* + 1). In this case, the metaheuristic algorithm determines the children by computing Equations (11)–(16). Thus, the particle swarm computes 84 operations for six parameters, and the metaheuristic algorithm computes 36 operations. In this case, the metaheuristic algorithm provides the population of each *k*-generation from crack topography through the parameters (*β*, *α*). Thus, the metaheuristic algorithm implements a suitable structure to obtain the next generation. The fourth examination is the selection, where the particle swarm selects the individual and global best. But, the metaheuristic algorithm selects the best parents and children. The fifth examination is the fitness error, where the particle swarm begins with a bigger error and slowly decreases. In this case, the particle swarm establishes the number of iterations since the beginning, interrupting the procedure at bigger errors. Instead, the error of the metaheuristic algorithm begins with a smaller error and decreases in moderated form. The error of the particle swarm is depicted in Figure 17 by the red line. Also, the error of the metaheuristic algorithm in every generation is depicted in Figure 17 by the black line.

Also, the metaheuristic algorithm can be examined based on the ant colony optimization regarding fitting accuracy and structure efficiency. In this case, the fitting accuracy of the ant colony is over 3% and computes more variables than the metaheuristic algorithm to determine crack contour models. Thus, the metaheuristic algorithm elucidates the viability to construct crack contour models with a relative error smaller than 1%. To corroborate these statements, the metaheuristic algorithm can be compared to the ant colony optimization of the crack contour models [*X_s_*(*u_r_*), *Y_s_*(*v_r_*), *Z_s_*(*w_r_*)], where the contour curves include wood surface, paper surface, metallic surface, surface with high reflectance, and texture surface. In this way, the ant colony structure is compared to the metaheuristic algorithm as follows. Thus, the first examination is the initial population, where the ant colony defines the range of the ants as a discrete set. Thus, the number of ants is six with a range of [0, 3], and their discrete values have the same probability. Instead, the metaheuristic algorithm determines the range of every weight by computing Equations (2)–(4) based on the surface topography. From this stage, the metaheuristic produces a smaller error in the first iteration. Also, the efficiency is elucidated by the search space, which provides the population near the optimal solution. This procedure reduces the number of iterations to construct the crack contour model. The second examination is the number of tested ants, where the ant colony initially defines the number of ants. This procedure leads to fall in a local minimum. Instead, the metaheuristic algorithm tests a new parent and a new weight in each generation through the mutation to avoid a local minimum. The third assessment is the population for the next iteration, where the ant colony computes the pheromone evaporation τ_new_ = (1-evaporation rate)τ_old_, the pheromone deposit (Deposit/Objective function), and a probability function *P*_i_ = τ*_i_*./Σ τ*_j_*. In this case, the metaheuristic algorithm determines the children by computing Equations (11)–(16). Thus, the ant colony computes 54 operations for six parameters, and the metaheuristic algorithm computes 36 operations. The fourth examination is the selection, where the ant colony selects the higher probability. But, the metaheuristic selects the best parents and children. The fifth evaluation is the fitness error, where the ant colony begins with a bigger error and decreases slowly. Instead, the error of the metaheuristic algorithm begins with smaller error and decreases in moderated form. The error of the ant colony is shown in Figure 17 by the green line.

Additionally, the metaheuristic algorithm is examined based on the simpler cubic B-splines. To do this, the B-splines generate the crack contour curves of the wood surface, paper surface, metallic surface, surface with high reflectance, and texture surface. Thus, the B-splines generate the contour curve through the expression *C*(*t*) = Σ*P_i_N_i_*_,3_(*t*), where *P_i_* contains the three-dimensional coordinates, *N_i_*_,3_ (*t*) are basis functions, and *t* is a point on the curve. In this way, the basis functions are computed by the expression *N_i_*_,3_(*t*) = (*t* − *u_i_*)(*N_i_*_,3-1_(*t*))/(*u_i_*_+3_ − *u_i_*) + (*u_i_*_+3+1_ − *t*)(*N_i+_*_1,3-1_(*t*))/(*u_i_*_+3+1_ − *u_i+_*_1_) [61]. Thus, the three-dimensional *B*-spline curve is computed by *Cx*(*t*) = Σ*x_i_N_i_*_,3_ (*t*), *Cy*(*t*) = Σ*y_i_N_i_*_,3_ (*t*), and *Cx*(*t*) = Σ*z_i_N_i_*_,3_ (*t*). The accuracy provided by the B-splines method is a relative error of 2.84%. Instead, the metaheuristic algorithm generates crack contour models, which represent crack surface contours with a relative error smaller than 1.0*%*.

Moreover, the metaheuristic algorithm is examined based on the least squares optimization as shown in Figure 2. To carry this out, the least squares method computes the weights of the crack contour curves [*X_s_*(*u_r_*), *Y_s_*(*v_r_*), *Z_s_*(*w_r_*)] of the wood surface, paper surface, metallic surface, surface with high reflectance, and texture surface. Thus, the least squares method computes the weights by minimizing the error between the crack coordinates and the Bezier functions, where the error is defined by the expressions e*_x_* = Σ[xi − x*_s_*(*u_i_*)]^2^, e*_y_* = Σ[*y_i_* − y*_s_*(*v_i_*)]^2^, e*_z_* = Σ[*z_i_* − z*_s_*(*w_i_*)]^2^. From these errors, the derivatives ∂e*_x_*/∂*w_i_* = 0, ∂e*_y_*/∂w_i_ = 0, ∂e*_z_*/∂*w*_i_ = 0 are computed to minimize the error. Expanding the summations of these derivatives, an equation system is obtained for each derivative, where each element of the equation system is a summation. Then, the systems of equations are solved to obtain the weights [*w_i_*, w*_i_*, *w_i_*]. Thus, least squares method computes the crack contour curves with a relative error of 3.167%, which is bigger than error of the metaheuristic algorithm. Additionally, the leas squares method performs more operations than the metaheuristic algorithm. This is because the systems of equations are deduced by summations.

Furthermore, the clothoids structure performs more operations than the Bezier curves through the cosine and sine functions. This is because the sine and cosine are determined by a summation of multiplications and divisions. Also, the curvature should be optimized to reduce the fitting error. But, the procedure to compute the curvature is performed by a great number of operations. Therefore, Bezier curves have been employed to optimize clothoids [62].

Additionally, the method based on peak–valley fails because the intensity profile does not provide the real surface topography. This criterion is elucidated by the surface of high reflectivity shown in Figure 13a, where the pick intensity is not located in the peak surface. Instead, the laser line projection depicts the real surface profile as shown in Figure 13b.

From these statements, the contribution of the crack contour modeling via the metaheuristic algorithm and micro-laser lane projection has been validated. Moreover, the simple setup increases the capability of the metaheuristic algorithm to perform crack contour modeling for surface quality inspection. This is because the arrangement includes only simple components such as a laser diode, CCD camera, slider device, and a computer. In this way, the proposed surface technique provides a contribution in the field of crack modeling and detection.

## 5. Conclusions

A technique to perform crack contour modeling by means of a metaheuristic algorithm and micro-laser line has been presented herein. The metaheuristic algorithm enhances crack contour modeling through the broken micro-laser line, which retrieves the crack topography. Thus, the crack contour model determines the crack region with great accuracy through the broken micro-laser line. This contribution is corroborated through the fitting accuracy and efficiency in optimizing the Bezier basis functions. The fitting accuracy contribution is achieved through the algorithm structure, which moves the Bezier curves toward the crack topography. This statement is validated by the results achieved in the crack contour modeling shown in Section 3, where the crack contour modeling provides a fitting accuracy of a relative error smaller than 1%. Also, the contour fitting accuracy includes the laser line scanning, which detects the crack region with great accuracy through the broken micro-laser line. Moreover, the algorithm’s efficiency is elucidated through the structure, which computes the control points through the crack surface in moderated time. Thus, the metaheuristic algorithm provides a valuable tool for performing crack contour modeling in the field of crack characterization and detection for surface quality inspection. In this way, the metaheuristic algorithm optimization via Bezier functions and laser line scanning has been performed to construct crack contour models in a good manner. In addition, the simple optical microscope system is available at a suitable cost to corroborate the capability of the crack contour modeling and detection. Thus, crack contour modeling can be carried out to good effect by employing a metaheuristic algorithm and micro-laser line scanning.

## Figures and Tables

**Table 1:** Contour control points generated via metaheuristic algorithm for the first generation.

Control Point	W_1,1_	W_2,1_	W_3,1_	W_4,1_	C_1,1_	C_2,1_	C_3,1_	C_4,1_	C_5,1_	C_6,1_
P_2_	8.6531	9.2428	12.1485	8.9334	9.4143	8.9626	8.6240	10.7138	10.8491	12.2839
P_2_	6.2754	5.5209	5.4768	5.6510	5.8795	5.9168	5.4836	5.5596	5.5682	5.6596
P_2_	6.3103	8.2724	6.6482	8.0516	7.2428	7.3399	6.2132	7.3152	7.3846	8.1211
P_3_	7.5245	8.1122	7.7542	9.8422	7.8038	7.8329	7.4954	8.7466	8.8499	9.9456
P_3_	5.6860	5.8692	5.7806	5.0125	5.7731	5.7821	5.6769	5.3776	5.4156	5.8186
P_3_	8.1652	7.5073	7.0306	5.7957	7.8200	7.8525	7.4748	6.3826	6.4437	7.0918
*Fitness*	2.3714	2.5324	2.1313	2.6918	2.4475	2.4427	2.5098	2.4062	2.3949	2.3088

**Table 2:** Contour control points generated via metaheuristic algorithm for the second generation.

Control Point	W_1,2_	W_3,2_	W_2,2_	W_4,2_	C_1,2_	C_2,2_	C_3,2_	C_4,12_	C_5,2_	C_6,2_	*n* = 103
P_2_	8.6531	10.6946	12.1485	12.2839	8.9342	8.9617	8.6256	10.7177	10.8452	12.2761	12.8107
P_2_	6.2754	5.3850	5.4768	5.6596	5.8806	5.9158	5.4857	5.5598	5.5680	5.6591	5.3214
P_2_	7.5245	6.8761	6.6482	8.1211	7.2456	7.3371	6.2188	7.3172	7.3826	8.1170	8.4953
P_3_	5.7532	8.0124	7.7542	9.9456	7.8047	7.8321	7.4971	8.7496	8.8469	9.9396	11.9875
P_3_	8.1652	5.7004	5.7806	5.8186	5.7733	5.7819	5.6774	5.3787	5.4145	5.8164	4.9751
P_3_	6.3103	6.3931	7.0306	7.0918	7.8209	7.8516	7.4766	6.3844	6.4420	7.0882	8.4786
*Fitness*	2.3692	2.3592	2.1313	2.3088	2.4474	2.4429	2.5094	2.4059	2.3952	2.3091	0.0002

**Table 3:** Microscope vision parameters and variables of the crack contour modeling.

Variable	Units	Value	Uncertainty	Variable with Uncertainty
*x_c_*	Pixels	395.9613	±0.004310	395.9613 ± 0.00431
*y_c_*	Pixels	373.3625	±0.005130	373.3625 ± 0.00513
*η*	Millimeter/pixels	0.0022	±0.000122	0.0022 ± 0.000122
*θ*	Deg	22.4316	±0.002260	22.4316 ± 0.002260
*d*_1_	Millimeters	62.3621	±0.001625	62.3621 ± 0.001625
*F*_1_	Millimeters	30.6889	±0.001232	30.6889 ± 0.001232
*d*_2_	Millimeters	60.2534	±0.001176	60.2534 ± 0.001176
*F*_2_	Millimeters	27.4037	±0.001083	27.4037 ± 0.001083
*z_i_*_,_ *_j_*	Millimeters			
*y_i_*_,_ *_j_*	Millimeters			
*x_i_*_,_ *_j_*	Millimeters			
x *_i_*_,_ *_j_*	Pixels			
y *_i_*_,_ *_j_*	Pixels			
*X_s_*(*u*)	Millimeters			
*Y_s_*(*u*)	Millimeters			
*Z_s_*(*u*)	Millimeters			
*w_r_*_+5_ *_s_*	Non-dimensional			
w *_r_*_+5_ *_s_*	Non-dimensional			
w *_r_*_+5_ *_s_*	Non-dimensional			
P *_r_*_+5_ *_s_*	Microns			
P *_r_*_+5_ *_s_*	Microns			
P *_r_*_+5_ *_s_*	Microns			
x *_i_*_,_ *_j_*	Pixels			
*I_i_*_,_ *_j_*	Gray level			
x(*u*)	Pixels			
*I*(*u*)	Gray level			

## Data Availability

All data supporting the findings are accessible by requesting a link or by e-mail to be added upon acceptance.
